# Spatially Confined/Ligand‐Directed Ultrabright NIR‐AIE Nanoprobe for In Vitro Rapid Detection and In Situ Imaging of Bacterial Septic Arthritis

**DOI:** 10.1002/advs.78156

**Published:** 2026-10-06

**Authors:** Yujian Li, Jia Shu, Sijia Lv, Leilei Qin, Xuefeng Luo, PengCheng Xiao, Huan Cai, Xin Gao, Yan Zhao, Haotian Zhou, Yaji Yang, Zhenglin Zhu, Shijia Ding, Juan Li, Rui Chen, Wei Huang

**Affiliations:** ^1^ Department of Orthopaedic Surgery The First Affiliated Hospital of Chongqing Medical University and Key Laboratory of Clinical Laboratory Diagnostics (Ministry of Education) Chongqing Medical University Chongqing China; ^2^ College of Laboratory Medicine Chongqing Medical University Chongqing China; ^3^ Chongqing Municipal Health Commission Key Laboratory of Musculoskeletal Regeneration and Translational Medicine Chongqing China; ^4^ Medical Examination Centre The First Affiliated Hospital of Chongqing Medical University Chongqing China; ^5^ Ophthalmology Medical Center The First Affiliated Hospital of Chongqing Medical University Chongqing Branch (Municipality Division) of National Clinical Research Centre for Ocular Diseases Chongqing China

**Keywords:** aggregation‐induced emissions, bacterial diagnostics, near‐infrared nanoparticles, photothermal therapy, point‐of‐care testing, real‐time bioimaging

## Abstract

Bacterial infection is a major threat to joint surgery and can lead to amputation or death. However, advanced tools for early, rapid, and accurate identification of joint‐infecting bacterial species remain limited. Here, we report a universal strategy for fabricating ultrabright near‐infrared (NIR) AIEgen fluorescent probes (PPANPs) that enable lateral flow assay (LFA)‐based rapid bacterial identification in joint fluid before surgery, as well as in situ imaging diagnosis and photothermal antibacterial intervention in the joint cavity. Structurally, PPANPs display a core–shell–shell architecture, in which aggregated NIR AIEgens form the fluorescent core, NIR lead sulfide (OA‐PbS) forms the inner shell, and poly(maleic anhydride‐alt‐1‐octadecene) (PMAO) forms the outer shell. This spatially confined design combines physical confinement, ligand‐mediated molecular organization, and cooperative luminescence within the PbS–AIE hybrid nanoassembly, promoting compact and ordered AIE packing and thereby enhancing NIR fluorescence intensity, quantum yield, and photothermal performance compared with conventional embedding methods. s a diagnostic platform, PPANP‐labeled LFA achieves 100% sensitivity for Staphylococcus aureus (*S. aureus*) identification within 10 min, outperforming standard plate culture (>96 h). Moreover, PPANPs enable rapid in situ imaging of *S. aureus* infection in mouse joints within 30 min and exhibit potent photothermal antibacterial activity in vitro under low‐power‐density laser irradiation.

## Graphical abstract

Inorganic PbS quantum dots spatially confine AIEgens and enable cooperative luminescence within a ligand‐directed core–shell–shell nanoassembly, generating ultrabright NIR PPANPs with enhanced fluorescence and photothermal performance. The platform enables 10‐min *S. aureus* identification in synovial fluid, in situ visualization of joint infection within 30 min, and strong photothermal antibacterial activity in vitro.

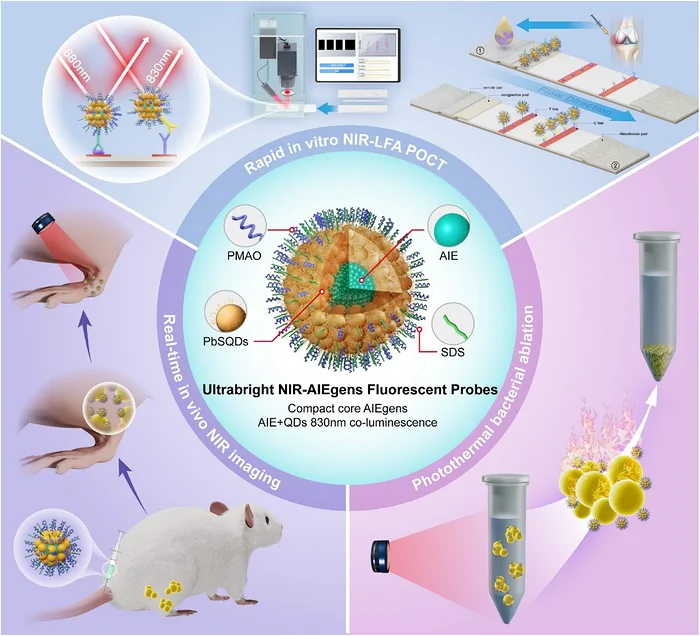

## 1 Introduction

Driven by global population aging, the prevalence of osteoarthritis has increased substantially, affecting over 595 million people worldwide and projected to exceed 1 billion by 2050 [1, 2]. With the rising burden of osteoarthritis leading to increasing demand for orthopedic surgeries, advances in surgical techniques and perioperative management have significantly improved patient outcomes [3, 4]. However, bacterial infections remain one of the most serious threats, with an incidence rate exceeding 4–10% of surgeries, leading to serious prognostic, life, and economic consequences for patients, including even death [5]. Current management of bone and joint infections, such as prosthetic joint infection (PJI), emphasizes rapid preoperative diagnosis and early in situ identification of the causative bacterial species, which can improve therapeutic outcomes and quality of life while reducing medical costs and mortality [6, 7]. Existing clinical diagnostic methods for bacterial infections of bone tissue mainly rely on bacterial cultures [8], polymerase chain reaction (PCR), mass spectrometry, and next‐generation sequencing (NGS) [9, 10, 11]. Nevertheless, these diagnostic approaches suffer from some obvious defects such as insufficient sensitivity, complex procedures, long detection times, expensive analytical instruments, and the need for skilled operators, which make it difficult to identify the bacterial species before surgery and in the initial infection stage, leading to the use of a large number of antibiotic therapies, increasing the resistance of patients and missing the optimal treatment window. Therefore, sensitive, simple, rapid, and low‐cost point‐of‐care testing (POCT) tools would be of key significance toward the identification and guidance of surgical protocols for bacterial infections.

For rapid diagnosis before surgery, LFA is now regarded as the most convenient POCT technology available for rapid on‐site screening of bacterial infections [12, 13]. Existing LFAs use signalling probes mainly colorimetric nanomaterials and fluorescent nanomaterials emitting in the visible region (400–650 nm) [14, 15]. Nevertheless, fluid samples from bacterial infections, such as joint fluids, usually have the following properties: (I) darker sample colors; (II) strong autofluorescence (400–650 nm) of small molecules produced by bacterial metabolism; and (III) low sample volume [16]. These properties make the conventional LFAs method prone to false‐positive or false‐negative results, making it difficult to accurately detect bacteria or bacterial metabolites in joint fluid. For intra‐articular diagnosis, fluorescence imaging is considered one of the most advanced strategies. However, the overlying musculoskeletal and soft tissues surrounding the joint cavity can significantly attenuate the emitted signal, leading to false‐negative results [17].

To counteract the above problems, a variety of near‐infrared (NIR) emitting fluorescent materials (700–1700 nm) have been developed as LFA and imaging signal probes to overcome the interference of absorption/autofluorescence in complex samples and the challenge of imaging penetration depth, including NIR quantum dots, conventional fluorescent dyes, aggregation‐induced emission (AIE) dyes, and their corresponding fluorescent nanoparticles [18, 19]. In contrast, NIR AIE dye‐based fluorescent nanoparticles have been recognized as a new type of promising NIR fluorescent probes owing to the large Stokes shift, good stability, and high brightness during aggregation. For example, in our previous work, we employed NIR‐AIE fluorescent nanoparticles as LFA signal probes to achieve sensitive and accurate quantification of targets in complex samples [20]. In addition, Tang et al. achieved in situ imaging of Mycobacterium tuberculosis in the lungs using NIR‐AIE fluorescent probes with polymer as the carrier [21]. However, NIR‐AIE fluorescent nanoparticles prepared by existing stencil swelling and polymer self‐assembly embedding methods face the challenge of a relatively low fluorescence quantum yield, which makes it difficult to meet the clinical needs of trace target analysis, limiting their applications. The main reasons are the low loading of AIE dyes by the organic solvent‐based stencil swelling (e.g., Polystyrene spheres) method and the inability to form a dense dye stacking structure inside the nanoparticles prepared by the polymer‐based (e.g., F127) self‐assembly method, resulting in the low luminescence intensity of the prepared AIE fluorescent nanoparticles [20, 22]. Furthermore, the low luminescence intensity implies that a high excitation light power is required, which will cause obvious photodamage. Thus, a new universal strategy for the preparation of NIR‐AIE fluorescent nanoparticles to enhance intermolecular aggregation and fluorescence intensity is urgently needed.

Here, we reported a universal liquid‐phase confinement cooperative self‐assembly strategy to fabricate new highly compressed and ordered ultrabright NIR‐AIEgens fluorescent nanoparticles (PPANPs) using alkane side chain (OA)‐modified amphiphilic polymers (PMAO) [23], OA‐functionalized NIR lead sulfide (OA‐PbS) quantum dots (QDs), and OA‐modified NIR‐AIEgens as building blocks (Figure 1a). PPANPs exhibited a typical core–shell–shell structure due to the driving force of OA ligands, where NIR AIEgens aggregated to form a fluorescent core, OA‐PbS QDs formed an inner shell, and a PMAO formed the outer shell. In this ordered spatial structure, the OA‐PbS inner shell promotes dense aggregation and ordered packing of AIE molecules through spatial confinement and ligand‐mediated molecular interactions, while the close integration of PbS QDs and AIEgens provides an additional cooperative luminescence contribution to the overall optical performance. The NIR fluorescence intensity, quantum yield, and photothermal performance of the obtained PPANPs were significantly higher than those of polystyrene sphere‐based stencil swelling and PMAO alone‐based self‐assembly embedding methods. Furthermore, the high fluorescent brightness and photothermal performance could be achieved with only low‐power‐density excitation light. The enhanced mechanism and interaction force of PPANPs with a core–shell–shell structure were systematically investigated by experiments and theoretical simulation. Using these outstanding properties, we further demonstrated the advanced applications of PPANPs in developing an LFA for rapid and accurate identification of bacteria in joint fluid before surgery (Figure 1b), as well as in situ early imaging diagnosis and therapy of bacteria in the joint cavity (Figure 1c). Thus, these PPANPs represent a universal approach to develop organic‐inorganic hybrid materials‐based ultrabright NIR‐AIEgens fluorescent probes with great translational potential. This is also the first reported case of their application for the early, rapid, and accurate identification and therapy of bacterial infections in orthopedic surgery.

**FIGURE 1:**
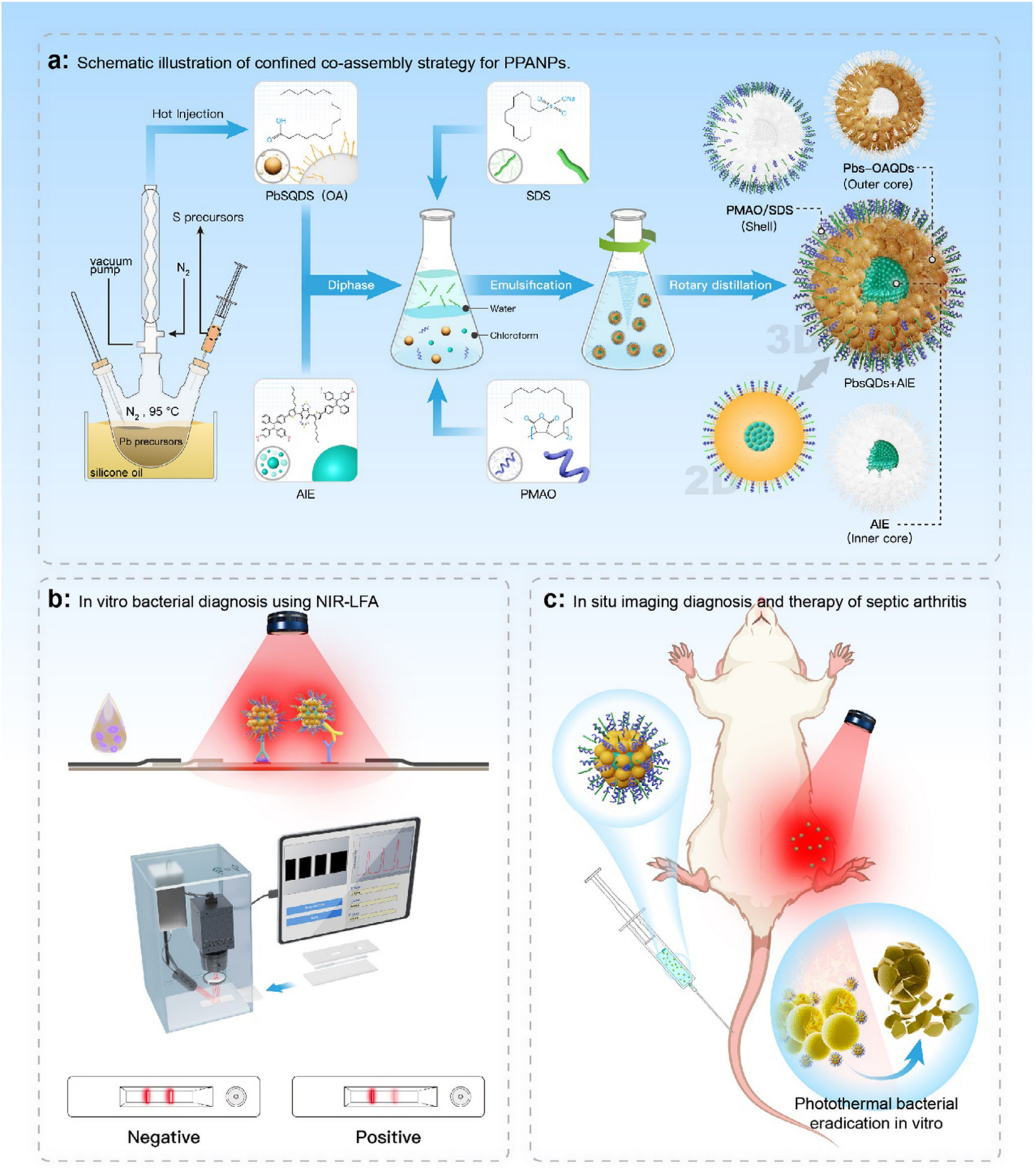
Schematic design and functional applications of PPANPs in bacterial diagnosis and therapy. (a) Schematic illustration of the confined co‐assembly strategy for constructing ultrabright NIR fluorescent nanoparticles (PPANPs) via co‐embedding PbS QDs and AIEgens in PMAO. (b) In vitro bacterial detection using PPANPs‐based NIR‐LFA in synovial fluid. (c) In situ NIR imaging diagnosis of septic arthritis; inset shows photothermal bacterial eradication in vitro.

## 2 Results

### 2.1 Synthesis and Characterization of Ultrabright NIR PPANPs

Ultrabright NIR PPANPs were synthesized by self‐assembly of hydrophobic PbS QDs and NIR AIEgens and PMAO as building blocks. As shown in Figure 1, a sodium dodecyl sulfate (SDS) aqueous solution was added to the chloroform solution containing hydrophobic PbS QDs, NIR AIEgens (Figure S1), and PMAO, followed by ultrasonic emulsification [24]. After removal of chloroform by rotary evaporation, the self‐assembled PPANPs were purified by centrifugation and resuspended in sodium hydroxide solution to hydrolyze the anhydride to form carboxyl groups. Hydrophobic PbS QDs containing a high concentration of oleic acid (OA) modification were prepared using the previously reported hot injection method [25]. The characterization results of the UV–vis absorption spectrum, fluorescence spectrum, and transmission electron microscopy (TEM) all verified the successful synthesis of hydrophobic PbS QDs (Figures S2 and S3). The obtained PbS QDs were used as inorganic components for the self‐assembly synthesis of PPANPs.

Considering that PbS QDs and NIR AIEgens affect the PMAO embedding effect, we first investigated the effect of different building block concentrations on the structure of the synthesised nanoparticles. As shown in Figure 2a–c, the synthesized PMAO‐PbS QDs complexes (PMAO@PbS) exhibited a uniform and regular core‐shell structure, and the PbS QDs were uniformly dispersed inside the PMAO shell. The particle sizes of the prepared PMAO‐PbS QDs complexes were 154, 194, and 242 nm when the concentration of PbS QDs was 6, 9, and 12 mg (Figures S4‐S6), respectively. Similar results were obtained for PMAO‐NIR AIEgens complexes (PMAO@AIE) (Figure 2d,e). The particle sizes of the PMAO@AIE were 192 and 264 nm when the AIEgens concentration was 1.5 and 9 mg (Figure S7), respectively. The above results indicated that PMAO embedded in both PbS and AIEgens could successfully form well‐defined core‐shell structures. Nevertheless, the 242 nm PMAO@PbS underwent aggregation and precipitation during storage mainly due to the high concentration of PbS QDs adsorbed on the surface of the composites (Figure S8), which increased the hydrophobicity. Therefore, 9 mg of PbS QDs‐based PMAO@PbS was used for subsequent self‐assembly experiments. According to the self‐assembly design strategy, we added different concentrations of NIR AIEgens to the reaction process of PMAO‐embedded PbS. As shown in Figure 2f–j, the resulting PPANPs exhibited a typical “core–shell–shell” structure, where the NIR AIEgens assembled into a compact core surrounded by dense hydrophobic PbS, and the outermost layer was coated with the PMAO shell. Consistently, DLS analysis showed that the hydrodynamic diameter of PPANPs increased with increasing AIEgen loading (Figure S9). The diameter of the NIR AIEgen core gradually increased from 72.28 to 145.65 nm as the AIEgen input increased from 0.25 to 1.5 mg, but remained nearly unchanged when the input was further increased to 2.0 mg, suggesting that, under the fixed PbS QD and PMAO contents, the effective confined space available for forming the compact AIE‐rich core approached its capacity at an AIEgen input of approximately 1.5 mg. Zeta‐potential analysis showed no obvious differences among the PPANP groups after AIEgen incorporation, indicating that the nanoparticles retained good colloidal dispersity (Figure S10). In contrast, PANPs lacking PbS showed reduced stability, suggesting a scaffold‐like role of PbS in maintaining nanoparticle uniformity and dispersion. The morphology of PPANPs and the corresponding spatial distribution of the PbS QDs and AIEgens were further verified by energy‐dispersive spectrometer (EDS) mapping and elemental line scanning spectra (Figure S11). The results in Figure 2k,l further displayed that the PbS QDs in PPANPs were uniformly distributed in the interfacial layer between the PMAO shell and the NIR AIEgen core, proving the successful synthesis of PPANPs.

**FIGURE 2:**
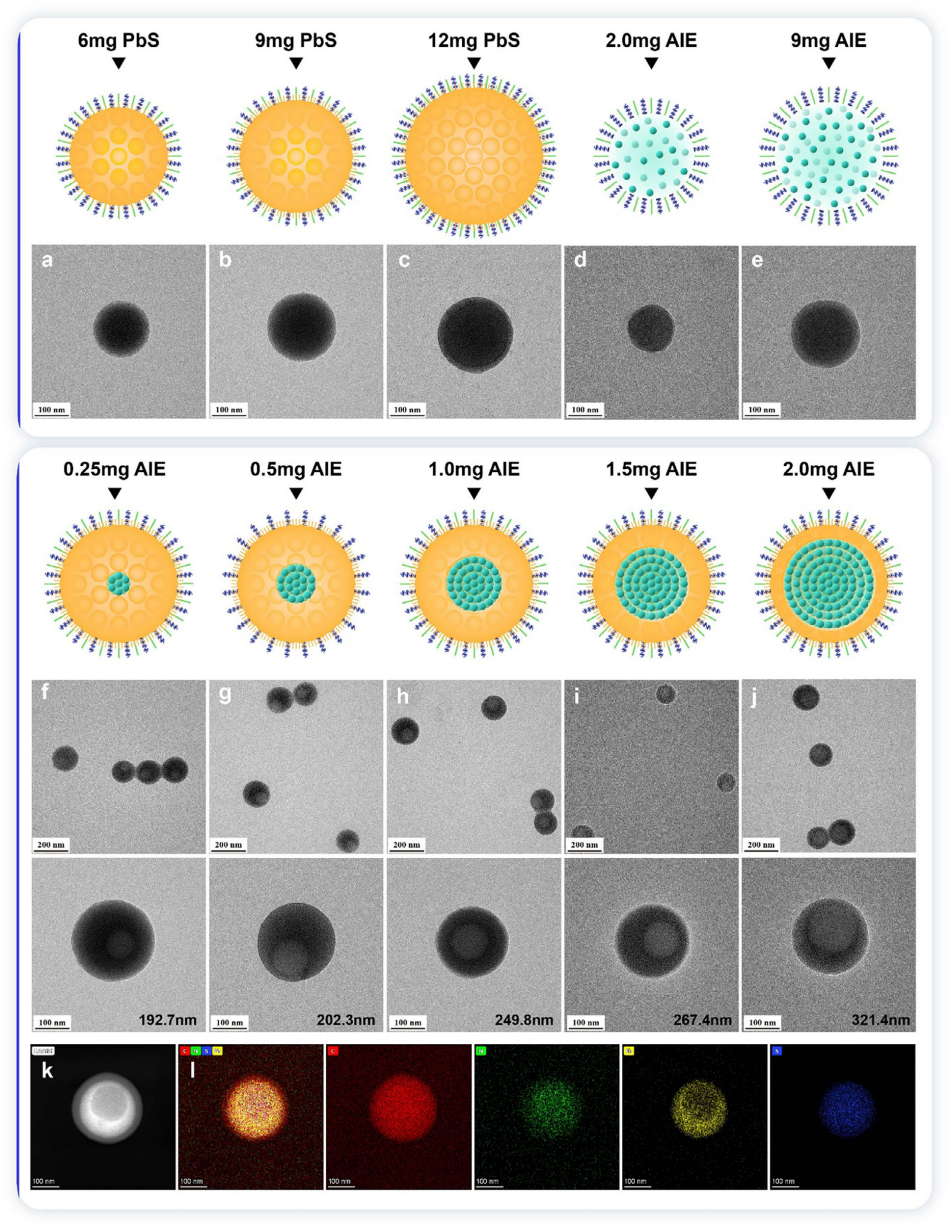
The morphology characterization of different PPANPs. (a–c) Structural diagram and TEM images (scale bar: 100 nm) of PMAO@PbS structures encapsulating different amounts of PbS QDs (6,9,12 mg). (d, e) Structural diagram and TEM images (scale bar: 100 nm) of PMAO@AIE structures encapsulating different amounts of AIE (1.5, 9 mg). (f–j) Structural diagram and TEM images (scale bars: 50, 100 nm) of PMAO@PbS@AIE structures encapsulating 9 mg of PbS QDs and varying amounts of AIE (0.25, 0.5, 1.0, 1.5, 2.0 mg). (k, l) HAADF‐STEM and EDS spectral analysis of PMAO@PbS@AIE.

The optical properties of nanocomposites are closely related to the structure, so we carefully characterized the UV absorption and fluorescence spectra of self‐assembled PPANPs. The results in Figure 3a (inset) showed that the solution color of the PPANPs gradually changed from brown‐black to dark green after using different concentrations of NIR AIEgen. The corresponding UV absorption spectra at 680 nm also increased by 243% (Figure 3a), indicating successful embedding of the AIE dye in the PMAO‐PbS QDs composite. The fluorescent intensity of the resulting PPANPs rapidly increased from 529.7 to 2594 a.u. as the AIEgens concentration increased from 0.25 to 1.5 mg, and then reached a plateau (2768 a.u.) as the concentration was further increased to 2 mg (Figure 3b). Thus, the 1.5 mg AIEgens‐based PPANPs were used for subsequent experiments. Figure 3c showed that the fluorescent intensity of the resulting PPANPs was 10 and 4 fold higher than that of pure 9 mg PbS‐based PMAO@PbS and 1.5 mg AIE‐based PMAO@AIE at the same nanoparticle concentration (Figure S12). Moreover, the fluorescent intensity was 2.1 fold brighter than that of 9 mg AIE‐based PMAO@AIE (Figure S13). We attributed the rapid increase in fluorescent intensity to the fact that the particle size of the prepared PPANPs did not increase compared to pure 9 mg PbS‐based PMAO@PbS (Figure 2i and Figure S5), but highly compressed the particle sizes of the AIE core and the PbS QDs shell layer (Figure 3e), significantly increasing the aggregation of the AIE dye. To further validate the optical performance of the PPANPs, we synthesized the PMAO‐encapsulated PbS QDs and FDA‐approved indocyanine green (PMAO@PbS@ICG) and polystyrene sphere (PS) encapsulated AIEgens (PS@AIE) (Figure S14) fluorescent nanoparticles as the controls (Figure 3d). The results showed that the fluorescent intensity of PPANPs was 3.7‐fold higher than that of PMAO@PbS@ICG at the same nanoparticle concentration. The relative quantum yield (QY) of PPANPs in aqueous solution was determined to be 16.4%, using water‐soluble ICG (QY  =  4.1%) as the reference (Figure S15) [26]. Moreover, the QY of PPANPs was 1.1 and 4‐folds higher than that of PS@AIE and ICG (Figure 3f). To further benchmark the optical performance of PPANPs against previously reported NIR‐AIE nanoprobes, representative polymer‐assisted and self‐assembled NIR‐AIE nanoprobes have exhibited quantum yields of up to 4.8% for amphiphilic block‐copolymer‐assembled NIR‐I AIE nanoparticles, 10.3% for PEGylated self‐assembled NIR‐II AIE nanoparticles, and 11.5% for DSPE‐PEG_2000_‐encapsulated NIR‐II AIE nanoparticles (Table S1) [27, 28, 29]. Although direct numerical comparison across different spectral windows and reference standards should be interpreted cautiously, the 16.4% quantum yield of PPANPs places them at the upper end of representative NIR‐AIE nanoprobes, supporting the effectiveness of the confined co‐assembly strategy in regulating aggregate‐state molecular packing and emission. The above results demonstrated that the obtained PPANPs had high fluorescent intensity and the developed novel core–shell–shell structure embedding strategy was significantly better than the pure polymer self‐assembly strategy and PS‐based template embedding strategy.

**FIGURE 3:**
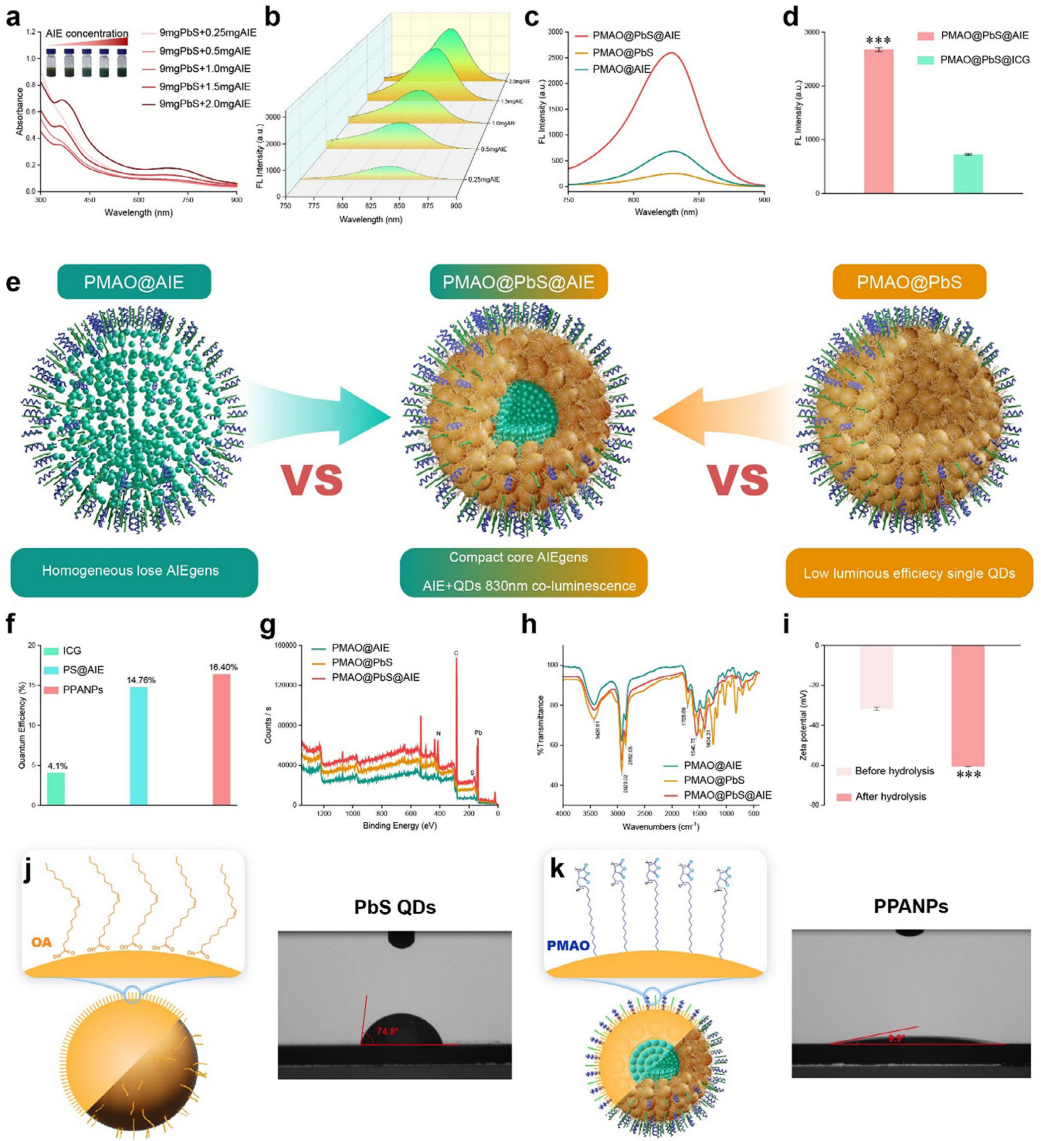
Characterization of PMAO@PbS@AIE PPANPs. (a, b) UV absorption and fluorescence spectra of PMAO encapsulating 9 mg of PbS QDs and varying amounts of AIE (0.25–2.0 mg). Insets show the physical photos of the solutions. (c) Fluorescence spectra of PPANPs, PPNPs, and PANPs at a concentration of 0.09 mg/mL. (d) Comparison of fluorescence intensity between PMAO@PbS@PMAO and PMAO@PbS@ICG(λem = 830 nm). (e) Schematic illustrations of the 3D structures of PPANPs, PPNPs, and PANPs. (f) QY of PMAO@PbS@AIE, PS@AIE, and ICG (dissolved in DW). (g, h) XPS and FT‐IR spectra of PPANPs, PPNPs, and PANPs. (i) Zeta potential of PPANPs before and after hydrolysis. (j, k) Molecular structure schematic and contact angle of PbS QDs and PPANPs. Data are presented as mean ± SD (n = 3), and statistical significance was determined using a two‐sided unpaired Student's *t*‐test. ^*^ *P* < 0.05, ^**^ *P* < 0.01, and ^***^ *P* < 0.001. In (d), significance is shown relative to PMAO@PbS@ICG; in (i), significance is shown relative to PPANPs before hydrolysis.

The X‐ray photoelectron spectroscopy (XPS) and X‐ray diffraction (XRD) peaks of PPANPs, PMAO@PbS and PMAO@AIE proved the coexistence of PbS QDs and AIEgens in the assembled PPANPs (Figure 3g and Figure S16). The Fourier‐transform infrared (FTIR) spectra (Figure 3h) of the PPANPs indicated a clear C−O bending at 1404.31 cm−1, which also confirmed that carboxyl groups were encapsulated on the PPANPs surface. The zeta potential of the obtained PPANPs before and after hydrolysis in NaOH solution decreased from −31.7 to −60.6 mV (Figure 3i), implying the successful hydrolysis of the anhydride in PMAO to carboxyl groups. Furthermore, the contact angle of pristine PbS QDs was 74.8°, while that of PPANPs decreased to 9.9°, confirming the successful transformation from a hydrophobic to hydrophilic surface (Figure 3j,k). To verify the potential for practical applications, we investigated the stability of PPANPs, including photostability, thermal stability, colloidal stability, acid‐base stability, fluorescent stability in various samples, long‐term storage stability, and oxidative stability under ROS‐mimicked conditions. All the results demonstrated the excellent stability of the prepared PPANPs (Figures S17–S24). All the aforementioned results collectively demonstrated the successful synthesis of PPANPs and proved that PPANPs could be used as a suitable NIR fluorescent reporter for LFA and imaging.

### 2.2 Molecular Dynamics Simulation of Fluorescence Enhancement Mechanisms

From the TEM images and dynamic light scattering (DLS) results, we observed that the particle size of the self‐assembled PPANPs did not change after the introduction of AIE at a fixed concentration of PMAO and PbS. AIEgens was confined by PMAO and PbS to form a fluorescent core inside the nanoparticles, resulting in a typical core–shell–shell structure and a significant enhancement of the fluorescent intensity. To elucidate how the molecular structures of PMAO, OA‐coated PbS, and AIE affect the self‐assembly behavior and the mechanism of fluorescence enhancement, we employed the molecular dynamics simulation strategy to simulate the self‐assembly and the formation mechanism of PPANPs and molecular interaction forces [30, 31]. Considering that the simulated molecular weight level was crucial for correlating simulations with experiments and obtaining high computational efficiency, we reported all variables and simulation parameters in reduced units. Herein, we have constructed three layered models in our simulations. As shown in Figure 4a, the first model demonstrated the interaction of OA‐coated PbS with AIE, with 100 AIE molecules (green spheres) embedded in the inner layer and OA‐coated PbS surrounding the outer layer (yellow spheres), with simulation box dimensions of 9.48 × 9.48 × 31.1 nm. The second model simulated the interaction of PMAO and AIE (Figure 4c). According to the TEM imaging calculation, the motion space of AIE molecules in PMAO@AIE was 8 times that of PPANP under the same particle size and fixed AIE concentration. Thus, the simulation box dimension of the second model (18.96 × 18.96 × 20 nm) was 8 times larger than that of the first model. Lastly, we established a control model with only AIE molecules (Figure 4e). After removing the PMAO layer, to maintain the same number of AIE molecules and motion space, the simulation box dimension was set to 18.96 × 18.96 × 14 nm. PACKMOL43 software was used to construct the initial configurations, and the CHARMM force field was employed to describe the system. The molecular force field consisted of nonbonded and bonded interactions [32]. The nonbonded interactions contained van der Waals (vdW) and electrostatic interactions, which were described by Equations 1 and 2, respectively. The vdW interaction of different kinds of atoms was based on the Lorentz−Berthelot mixing rules (Equation 3), and the cutoff distance of the interaction between vdW and electrons was set at 1.2 nm. The particle mesh Ewald (PME) method was employed to calculate the long‐range electrostatic interactions. In our simulation, energy minimization was employed to relax the simulation box, and a canonical (NVT) ensemble with a 1.0 fs time step was used to optimize the simulation box. To ensure that the system reached a stable state, the temperature was maintained at 300 K through the Nose‐Hoover thermostat; the optimization time was set to 20 s, and the trajectory coordinates of the molecules were collected at a storage frequency of 20 000 steps. The motion of atoms in the simulation of atomic dynamics was described by the classical Newton equation and solved by the velocity‐Verlet algorithm. All simulations were performed with the GROMACS 2019.6 package [33].

(1) $$E_{LJ}\;\left(\gamma_{ij}\right)=4\varepsilon_{ij}\left({\left(\frac{\varepsilon_{ij}}{r_{ij}}\right)}^{12}-{\left(\frac{\varepsilon_{ij}}{r_{ij}}\right)}^{6}\right)$$

(2) $$E_{c}\;\left(r_{ij}\right)=\frac{q_{i}q_{j}}{4\pi \varepsilon_{o}\varepsilon_{r}r_{ij}}$$

(3)

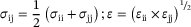




**FIGURE 4:**
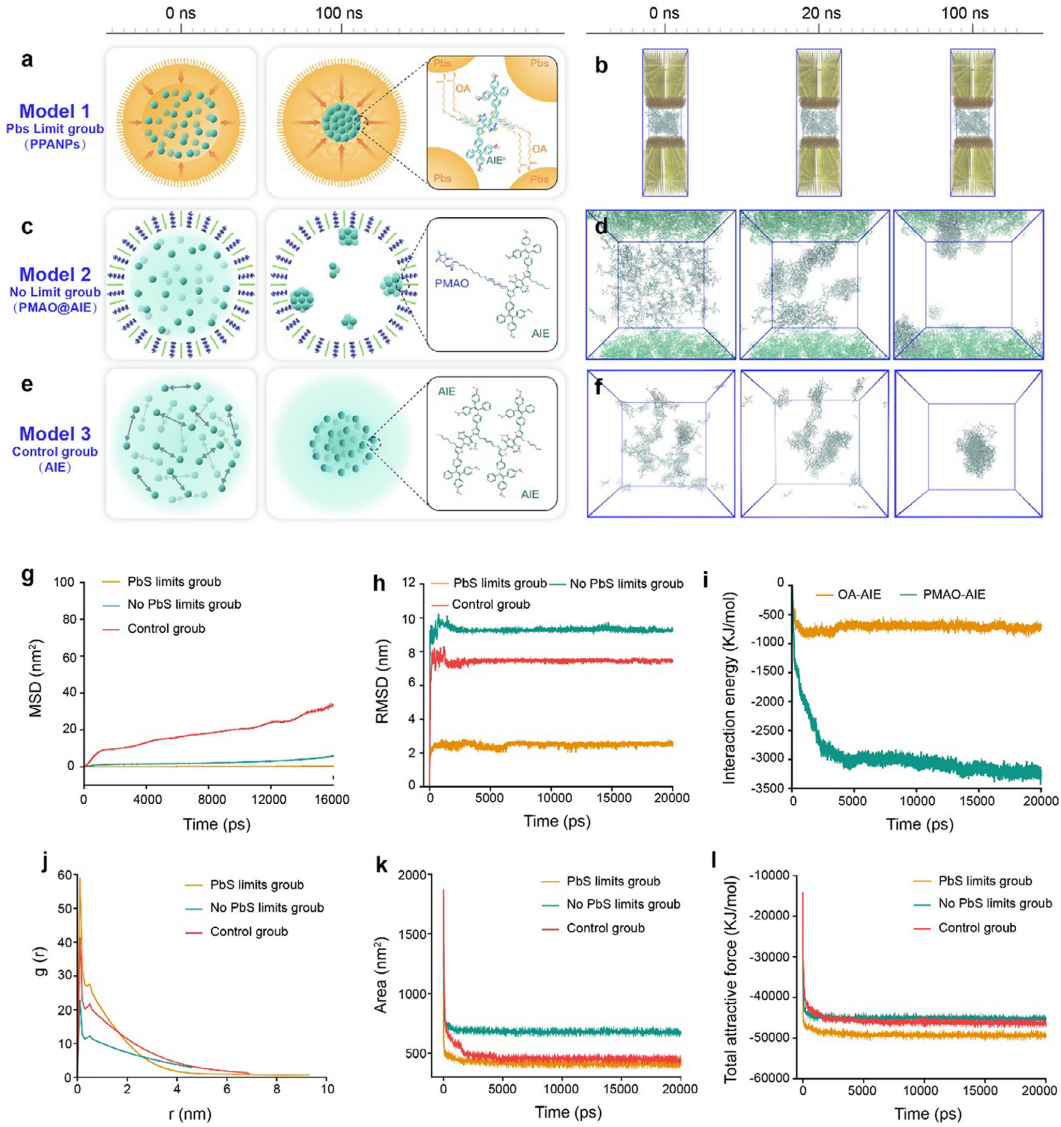
Molecular dynamics simulation of the AIE aggregation and fluorescence enhancement mechanism in PPANPs with PbS QDs and the PMAO spatial confinement model. (a, b) Schematic illustration of the PbS QDs spatial confinement model for AIE and the simulation equilibrium process (Model 1). The inset shows the interaction between the OA ligand on the PbS surface and AIE molecules. (c, d) Schematic illustration of the PMAO spatial confinement model for AIE and the simulation equilibrium process (Model 2). The inset shows the interaction between the amphiphilic polymer PMAO and AIE molecules. (e, f) Schematic illustration of the AIE self‐aggregation model and simulation equilibrium process (Model 3). The inset shows the interaction between AIE molecules. (g) Mean square displacement (MSD) of the central carbon atoms on AIE in different models. (h) Root mean square deviation (RMSD) of the central carbon atoms on AIE in different models. (i) Analysis of the interaction forces between PAMO, OA ligands, and AIE molecules (Coulomb (SR) + Lennard‐Jones (SR)). (j) Radial distribution function (RDF) of AIE molecules in different models. (k) Solvent‐accessible surface area (SASA) of AIE molecules in different models. (l) Analysis of the interaction forces between AIE molecules in different models (Coulomb (SR) + Lennard‐Jones (SR)).

In the three simulated models, AIE molecules uniformly distributed in the simulated space showed a tendency of spontaneous aggregation. As shown in Figure 4a,b, AIE molecules in Model 1 were fixed in the core and highly aggregated during the 0–100 ns movement time, due to the spatial limitation of PbS‐OA and the surface ligand affinity. In Model 2 (Figure 4c,d), AIE molecules first formed polydisperse clusters within 0–20 ns, and then gradually converged into larger clusters as the movement time increased to 100 ns, due to the molecular interaction between PMAO and AIE. In Model 3 (Figure 4e,f), AIE molecules first formed polydisperse clusters within 0–20 ns, and gradually converged into a large loose cluster as the movement time increased to 100 ns, due to the hydrophobic interaction between AIE molecules.

To further investigate the molecular mechanism of the three models, we characterized the intermolecular interaction parameters. Figure 4g,h exhibited the mean square displacement (MSD) and root‐mean‐square deviation (RMSD) of AIE molecules in different models, which could evaluate the motion range of molecules or atoms over time, as well as the mean deviation of the position of molecules or atoms from the reference structure during the simulation process, reflecting the aggregation ability of AIE molecules. Based on the Einstein relation, we calculated the diffusion coefficient from the slope of the MSD‐time relationship. This coefficient not only reflects the rate of molecular motion in a specific environment but also reveals the degrees of freedom in molecular motion and potential dynamic constraints. The MSD value of model 1 was significantly lower than that of model 2 and model 3 (Figure 4g), and the corresponding diffusion coefficients were 0.0023, 0.067, and 0.3505, respectively, indicating that AIE molecules had enhanced aggregation ability and decreased diffusion ability under the spatial constraint of PbS‐OA. A low RMSD value indicated less change in position and a more stable structure, while a high RMSD value indicated a larger change in structure caused by folding, rotation, or aggregation. As shown in Figure 4h, the RMSD value of Model 1 was significantly lower than that of Model 2 and Model 3 after simulation, indicating that the limited space inside the PbS‐OA QDs promoted a tighter and more stable arrangement of AIE molecules. The primary cause for Model 1 having a stronger compression capability than Model 2 lies in the fact that the attraction of the amphiphilic polymer PMAO toward AIE molecules is greater than that of PbS‐OA QDs to AIE molecules (Figure 4i), resulting in the dispersed aggregation of AIE molecules in PMAO. Furthermore, the large number of OA ligands on the PbS surface can stably fix the OA side chains of the AIE molecule, which is beneficial for the arrangement and aggregation of the AIE molecule. The above results showed that the model 1 structure was more favorable for the aggregation of AIE molecules. The degree of aggregation of AIE molecules was further elucidated through radial distribution function (RDF), solvent‐accessible surface area (SASA), and total attractive force (TAF) = Coulomb (SR)+ Lennard‐Jones (SR). The RDF described the probability of finding another AIE molecule within a certain distance, reflecting the distance between molecules. As shown in Figure 4j, the first RDF peak appeared at 0.1 nm for Model 1, which was significantly lower than that of Model 2 (0.2 nm) and Model 3 (0.2 nm), indicating a higher compression degree of AIE molecules in Model 1. The SASA measures the surface area of AIE molecules in the aggregated state. As shown in Figure 4k, under the compression of PbS‐OA QDs, the AIE molecule surface area in Model 1was significantly lower than that of Model 2 and Model 3, indicating that Model 1 had the highest compression ability for AIE molecules. The total gravitational force reveals the *π–π* stacking and aggregation behavior of the AIE molecule [34, 35]. As shown in Figure 4l and Figure S25, the TAF between AIE molecules in Model 1 (−32677.54 KJ/mol) was greater than that in Model 2 (−26166.73 KJ/mol) and Model 3 (−18208.88 KJ/mol), indicating that the AIE molecules effectively realized π‐π stacking under the compression of PbS‐OA QDs and the interaction with OA ligands, thereby enhancing aggregation and luminescence intensity [36]. The dihedral angle distributions of the AIE side chains in all three models exhibited consistent peak positions at approximately −136°, −49°, 51°, and 149°, indicating that AIE molecules maintained a stable conformation irrespective of spatial confinement or hydrophobic interactions (Figure S26). Taken together, these results indicate that the OA‐PbS inner shell contributes to fluorescence enhancement through multiple but distinguishable mechanisms. First, spatial confinement by the PbS‐OA layer promotes tighter AIE packing and restricts molecular motion, consistent with the restriction‐of‐intramolecular‐motion mechanism of AIE, in which suppression of molecular motion reduces nonradiative relaxation and favors radiative emission [32]. Second, the OA ligands on the PbS surface interact with the alkyl side chains of the AIE molecules, facilitating their organization and stabilization within the confined region, consistent with previously reported ligand‐directed regulation of AIEgen–inorganic nanoassemblies [23]. In addition to these structural effects, the markedly stronger fluorescence of PPANPs compared with the corresponding PbS‐ or AIE‐containing control nanoparticles suggests an assembly‐level cooperative luminescence contribution arising from the close integration of PbS QDs and AIEgens. However, the present steady‐state optical data are insufficient to establish a specific PbS‐to‐AIE energy‐transfer pathway. Therefore, cooperative luminescence is retained as an overall optical contribution of the hybrid nanoassembly, whereas direct intercomponent energy transfer is considered a possible but currently unconfirmed mechanism.

### 2.3 In Vitro Diagnosis of *S. Aureus* using PPANPs‐Labeled LFA Platform

*S. aureus*, the most prevalent pathogen in osteoarticular infections and an independent risk factor for infection recurrence, secretes the exotoxin Hla (α‐hemolysin), which plays a critical role in cytotoxicity and bacterial colonization [37, 38]. However, there is currently no available method for POCT of *S. aureus*. For accurate quantitative detection, we introduced chicken IgY‐modified PPANPs and goat anti‐chicken IgY antibodies as an independent Control (C) line signaling system for the ratiometric measurement (Figure 5a). After directly adding the *S. aureus*‐negative joint fluid sample, the PPANPs@Ab probes directly reacted with the Hla protein immobilized at the T line to form the immune complex, resulting in the simultaneous appearance of fluorescent bands at both the T line and the C line. After the reaction was completed (10 min), the fluorescence of the T line and C line on the LFA test strip was read using a laboratory‐built portable miniaturized NIR LFA instrument (20 cm × 6.5 cm × 10 cm). As shown in Figure 5b, the NIR signal reader was composed of two 680 nm excitation lasers with matched output power, a CMOS camera, and various optical components. The two lasers were positioned to illuminate the fixed T‐line and C‐line regions, respectively. After installing the corresponding software App, the captured NIR fluorescence signals could be output in real time via wired or wireless means, and the ratio of the fluorescence intensity of the T line to that of the C line (FI_T_/FI_C_) could be calculated. To validate the accuracy of the custom‐built device, we compared its signal output with results from a commercial fluorescence spectrometer across all previously constructed samples, and found excellent agreement (Figure S27), thereby confirming its reliability for subsequent experiments.

**FIGURE 5:**
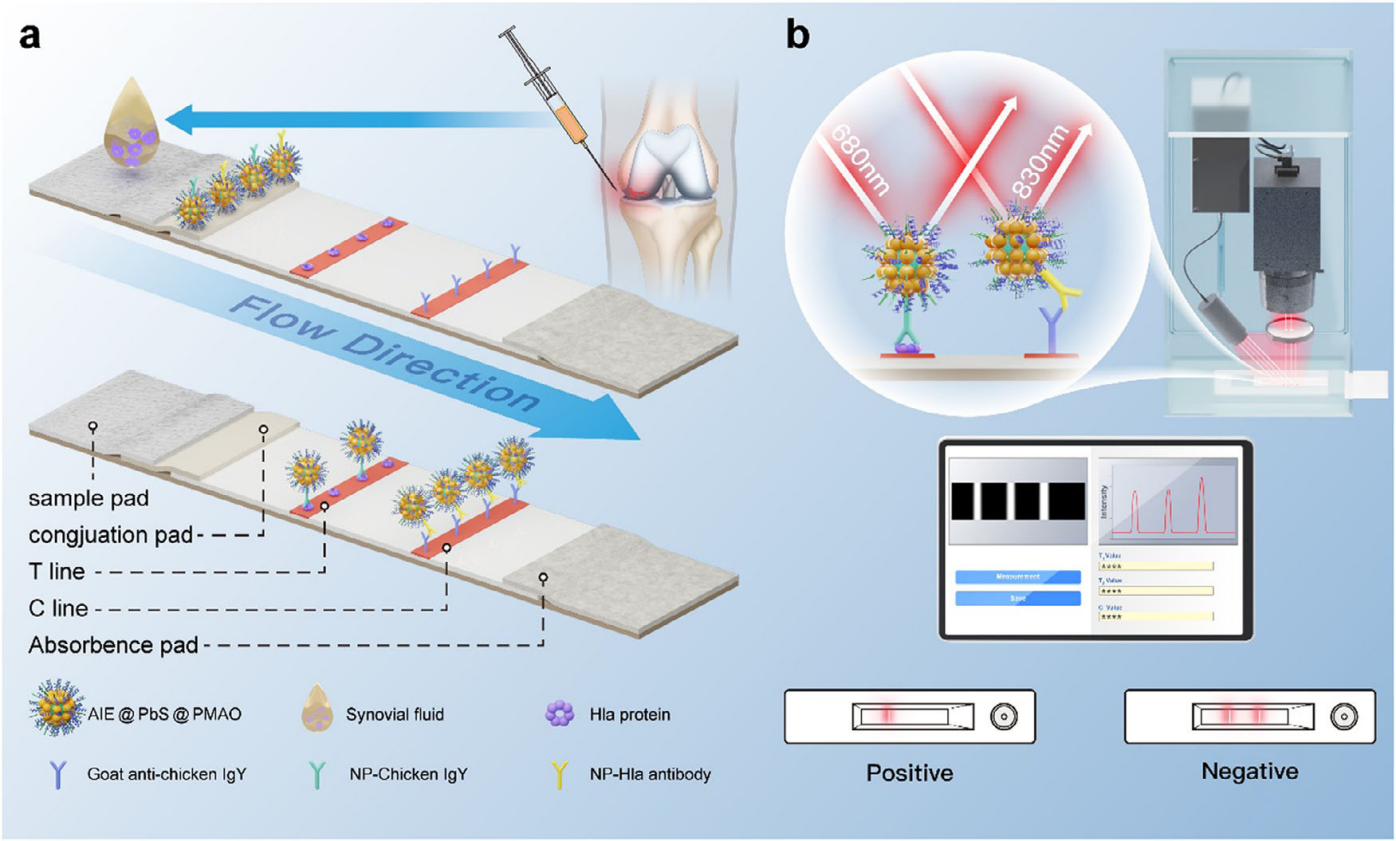
Principle of the PPANPs‐based NIR‐LFA system. (a) Mechanism of the PPANPs‐based NIR‐LFA for ultrasensitive POCT detection of *S. aureus*–induced septic arthritis. (b) Working principle of the laboratory‐built portable miniaturized NIR‐LFA instrument.

Synovial fluid samples from bacterial and non‐bacterial infections were characterized by deep color, high content of protein/small molecule substances, and small sample volume (Figure 6a), which could cause severe absorption (Figure 6b) and autofluorescence interference (Figure 6c) to the fluorescence of detection probes, resulting in poor diagnostic sensitivity and precision [39]. Moreover, the problems mentioned above could not be effectively solved by centrifugation and continuous dilution. To verify the effectiveness of directly detecting the target in untreated joint fluid samples, we investigated the fluorescence intensity changes of the synthesized NIR PPANPs probes in the samples, and used green, orange, and red luminescent quantum dots as the control group (Figure S28). As shown in Figure 6d, the fluorescent intensity of visible light quantum dots (green, yellow, and red) decreased by more than 99.7%, while the fluorescent intensity of the PPANPs hardly decreased, indicating that the NIR PPANPs could eliminate the interference of absorption and autofluorescence. Considering the need to detect NIR fluorescence signals on nitrocellulose (NC) membranes, we further verified the absorption and autofluorescence of the samples on the NC membrane, as well as the changes in fluorescent intensity of the PPANPs after contact with the samples on the membrane. As shown in Figure 6e, 0.5 µL of BBS buffer and synovial fluid samples of different dilution ratios were spotted on the NC membrane, and a dry NC membrane was used as the control. For the NIR LFA instrument detection, it was found that neither the dry NC membrane nor the NC membrane covered with samples detected any fluorescence signals (Figure 6f). For the visible‐region LFA instrument detection, no fluorescence signal was detected on the dry NC membrane, while the NC membranes covered with BBS buffer and joint fluid samples showed obvious scattered fluorescence and strong light absorption capacity (Figure 6g). After the quantum dots and PPANPs were mixed with the diluted joint fluid samples and dropped onto the NC membrane (Figure 6e), detected fluorescence signal of the visible‐region quantum dots was almost completely attenuated, whereas the fluorescence signal of PPANPs showed only minor fluctuations (Figure 6h), indicating that NIR detection essentially avoided the interference caused by sample absorption, autofluorescence, and light scattering. The above results indicated that PPANPs were suitable as a detection probe for LFA to directly detect targets in untreated joint fluid samples.

**FIGURE 6:**
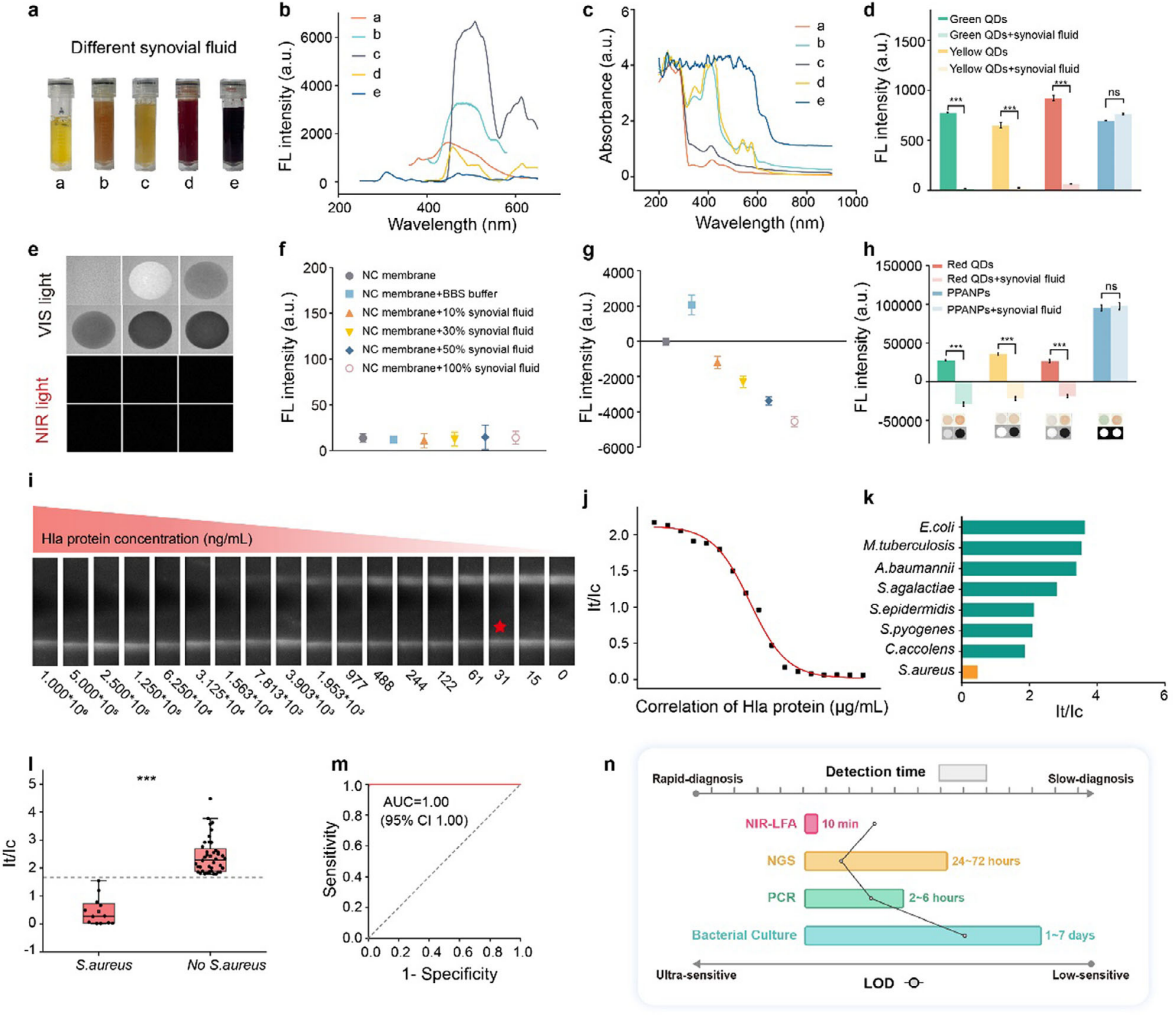
Background interference of synovial fluid and clinical validation of *S. aureus* detection using the NIR‐LFA platform. (a) Photograph of synovial fluid samples. (b) UV–vis–NIR absorption spectra of synovial fluid. (c) Fluorescence spectra of synovial fluid samples. (d) Fluorescence intensity of visible‐region quantum dots and NIR fluorophores in aqueous solution and synovial fluid. (e) Photographs of NC membranes loaded with different concentrations of synovial fluid captured under visible and NIR light. (f) NIR fluorescence signals of different concentrations of synovial fluid on NC membranes. (g) Visible‐region fluorescence signals of different concentrations of synovial fluid on NC membranes. (h) Fluorescence intensity changes of visible‐region quantum dots and PPANPs before and after loading synovial fluid samples, captured using the portable NIR‐LFA instrument (inset shows captured images on the NC membrane). (i) NIR fluorescence images of NC membranes loaded with different concentrations of Hla protein, captured by the portable NIR‐LFA instrument. (j) Sensitivity curve for Hla protein detection. (k) Specificity of NIR‐LFA for detecting different bacterial species. (l) Box plot of clinical synovial fluid samples from *S. aureus*‐infected patients. (m) AUC curve for detecting *S. aureus* infection in clinical synovial fluid samples. (n) Comparison of NIR‐LFA with bacterial culture, PCR, and NGS methods.

To achieve good detection performance of the developed test strip, we comprehensively optimized the experimental conditions, including the immunoreaction time, PPANPs@Ab probe concentration, the exposure index of the CMOS camera, and the Hla protein immobilized concentration on the T line (Figures S29–S32). Under the optimal conditions, the detection performance of the PPANPs‐LFA was investigated by analyzing a range of joint fluid samples with different Hla concentrations (0–10^6^ ng mL^−1^). Figure 6i revealed that with decreasing Hla amount, the fluorescent band on the T line gradually brightened under a 680 nm laser (1 W/cm^2^), confirming the competitive immunoassay principle. The corresponding FI_T_/FI_C_ also gradually increased as the Hla concentration decreased (Figure 6j) and the linear detection range for Hla was 31–1.25×10^6^ ng mL^−1^(y = 0.01+1.56/(1+x/2.44)^1.02^, R^2^ = 0.99). Accordingly, the limit of detection (LOD), defined as the average FI_T_/FI_C_ value of negative samples minus three times the standard deviation, was as low as 31 ng mL^−1^ for Hla. Importantly, the assay maintained a clear concentration‐dependent response in the low‐Hla range approaching the LOD, demonstrating sensitive detection performance under weak‐positive, low‐target conditions relevant to low‐level and early‐stage infection. The specificity of the LFA was evaluated by testing synovial fluid samples after infection with other bacteria, including Escherichia coli, Mycobacterium tuberculosis, Acinetobacter baumannii, Streptococcus agalactiae, Staphylococcus epidermidis, Streptococcus pyogenes, and Corynebacterium accolens. Figure 6k indicated the lowest FI_T_/FI_C_ toward *S. aureus*‐positive samples (0.449) compared with those toward other bacterial infection samples, suggesting good selectivity. The practicability of the PPANPs‐LFA was investigated using 48 clinical synovial fluid samples included in the final analysis, comprising 12 *S. aureus*‐positive and 36 *S. aureus*‐negative samples. ROC curve analysis based on the clinical FI_T_/FI_C_ values was performed to determine the optimal diagnostic cutoff, which was selected by maximizing the Youden index (sensitivity + specificity − 1). The optimal FI_T_/FI_C_ cutoff was determined to be 1.664, and samples with FIT/FIC values below 1.664 were classified as *S. aureus*‐positive (Table S2 and Figure S33). Patients were classified as PJI or aseptic failure according to the 2018 Musculoskeletal Infection Society criteria, and pathogen identification was confirmed by prolonged synovial fluid culture together with postoperative histopathological examination (Table S3). As shown in Figure 6l, all 12 *S. aureus*‐positive samples and all 36 *S. aureus*‐negative samples were correctly classified within 10 min, corresponding to a sensitivity, specificity, PPV, NPV, and overall accuracy of 100% (Table S4). ROC analysis further yielded an area under the curve (AUC) of 1.0 (Figure 6m). We further verified the reliability of the PPANPs‐LFA by simultaneously testing 12 *S. aureus*‐positive samples through the plate (TSA medium) culture method (gold standard method). After 72 h of culture, only 6 joint fluid samples grew colonies (Figure S34). Considering that *S. aureus* has the intracellular infection ability, the 6 samples without colonies were subjected to ultrasonic therapy and then continuously cultured for 96 h. Results in Figure S31 showed that *S. aureus* colonies were cultivated from all samples. In other words, the PPANPs‐LFA could achieve the effect of 168 h of plate culture in just 10 min, and also avoid false negative results caused by intracellular infection of *S. aureus*. Moreover, the detection time of the developed PPANPs‐LFA method was also significantly better than that of the PCR and NGS methods (Figure 6n). The above results indicated that the constructed PPANPs‐LFA strategy was highly suitable for the rapid and accurate diagnosis of *S. aureus* infection before PJI surgery, providing a basis for the surgical and medication plans of patients [40].

In addition, the universality of the developed PPANPs‐LFA strategy for directly detecting untreated joint fluid samples was verified by detecting C‐reactive protein (CRP) in the joint fluid (Figures S35–S38). The linear detection range for CRP was 0.39‐200 µg mL^−1^ (y = 5.01×(1‐e^(‐0.03854×x)^), R^2^ = 0.97), with a LOD of 0.39 µg mL^−1^, which could meet the clinical needs for diagnosing acute infections (100 µg mL^−1^). Furthermore, the quantitative CRP detection results of 52 joint fluid samples with bacterial infections showed a good correlation with the chemiluminescence (diluted for testing) detection results (Figure S39). The above results indicated that the constructed PPANPs‐LFA strategy had excellent detection performance and good universality.

### 2.4 In Vivo Imaging and Photothermal Antibacterial Performance of PPANPs

In the early stage of bacterial infection in the joint, patients only experience slight joint pain, and no large amount of joint fluid is produced in the joint cavity, making it difficult to achieve in vitro diagnosis by extracting joint fluid [41, 42]. Therefore, achieving in situ bacterial diagnosis and therapy in the early stage of joint bacterial infection can significantly improve the prognosis and reduce the economic burden on patients [43]. To address the above challenge, we developed a theranostics probe by modifying PPANPs with anti‐*S. aureus* antibodies (PPANPs@sAb), enabling in situ early diagnosis and photothermal therapy of *S. aureus* in the joint cavity [44]. As shown in Figure S40, the results of particle size and zeta potential proved that the theranostic probe was successfully prepared. To verify the binding ability of the diagnostic probe, we constructed *S. aureus* capable of expressing green fluorescent protein (GFP) [45] through genetic engineering and observed the fluorescence signal by Confocal Laser Scanning Microscopy (CLSM) (Figure 7a). CLSM images showed that an obvious colocalized fluorescent signal (red) of PPANPs appeared on the surface of the *S. aureus* (green) (Figure 7b), while no such signals were observed in the control group (Figure 7c). The overlap of red and green fluorescence signals in the experimental group was further confirmed by fluorescence intensity profile analysis (Figure 7d,e), indicating the targeting ability of the theranostic probe to *S. aureus*. As PPANPs@sAb could effectively target bacteria in vitro, the in situ diagnostic capability for *S. aureus* at the infection site was further investigated in a mouse model of joint infection induced by *S. aureus* (Figure 7f). At 24 h after intra‐articular inoculation with *S. aureus* (1 × 10^6^ CFU), PPANPs@sAb was administered intravenously, followed immediately by NIR fluorescence imaging. A distinct fluorescence signal at the infected joint was observed as early as 30 min after probe administration (Figure 7g), whereas almost no fluorescence signal was detected in the corresponding joint region of uninfected mice (Figure 7h). Notably, the fluorescence intensity at the infection site further increased from 60 to 120 min after PPANPs@sAb administration, while the control group remained at a low fluorescence level, indicating effective infection‐site targeting and good specificity (Figure 7i). These results demonstrate that PPANPs@sAb enables rapid in situ visualization of *S. aureus* joint infection within 30 min after probe administration.

**FIGURE 7:**
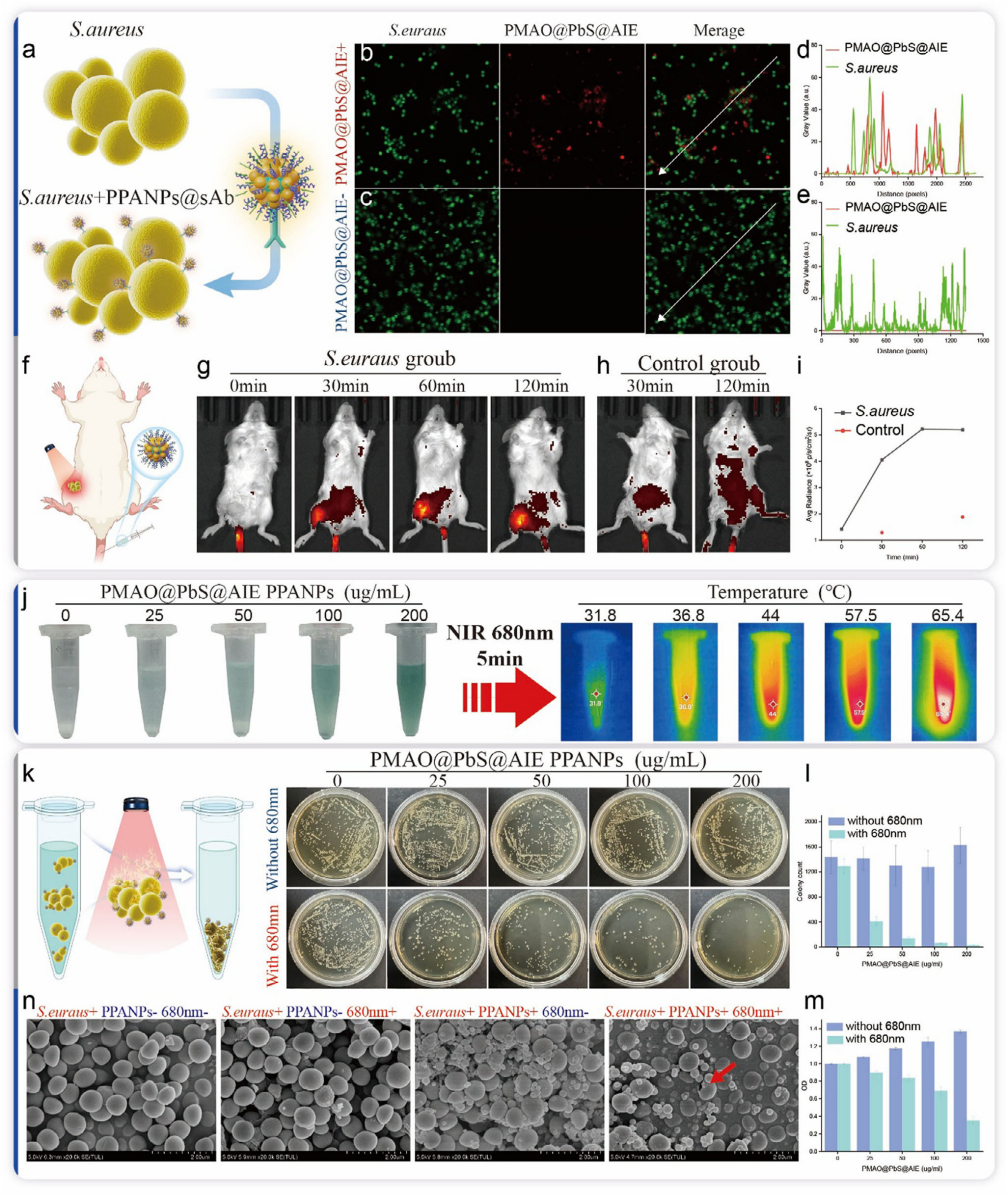
Colocalization‐based NIR imaging capability and antibacterial activity analysis of PPANPs@sAb targeting *S. aureus*. (a) Schematic diagram illustrating the targeted binding of PPANPs@sAb to *S. aureus*. (b, c) Confocal images showing the colocalization of PPANPs@sAb (red) with GFP‐labeled *S. aureus* (green). (d) Fluorescence intensity profiles of the linear region along the white arrow in b. (e) Fluorescence intensity profiles of the linear region along the white arrow in c. (f) Schematic illustration of PPANPs@sAb‐based in situ imaging of *S. aureus* infection within the mouse joint cavity. (g) Time‐dependent NIR imaging of mice with intra‐articular *S. aureus* infection in the supine position after intravenous injection of PPANPs@sAb. (h) Time‐dependent NIR imaging of control mice without *S. aureus* infection in the joint cavity in the supine position after intravenous injection of PPANPs@sAb. (i) Time‐dependent changes in NIR fluorescence intensity in the joint cavity of mice shown in g and h. (j) Temperature elevation of PPANPs at different concentrations after 680 nm laser irradiation (1 W/cm^2^,5 min). (k) Schematic illustration of the photothermal antibacterial effect of the PPANPs@sAb and *S. aureus* mixture under NIR irradiation in an Eppendorf tube, along with photographs of bacterial colonies on LB agar plates after treatment with different concentrations of PPANPs@sAb. (l) Bacterial viability after treatment with different concentrations of PPANPs@sAb was quantitatively analyzed using the plate counting method. Data are presented as mean ± standard deviation (n = 3). (m) OD600nm values of *S. aureus* suspensions after photothermal treatment with different concentrations of PPANPs@sAb. Data are presented as mean ± standard deviation (n = 3). (n) Representative HRTEM images of *S. aureus* after different treatments. Red arrows indicate ruptured *S. aureus* cells.

Considering the outstanding photothermal performance of PbS and aggregated AIE molecules, we further investigated the photothermal antibacterial efficacy of the PPANPs [21, 46]. We first investigated the photothermal performance of PPANPs at various concentrations. As shown in Figure 7j, the color of the PPANPs solution changed from colorless to dark green as the PPANPs concentration increased from 0 to 200 µg mL^−1^. After being exposed to a 680 nm laser (1 W/cm^2^), the PPANPs solution temperature dramatically increased from 31.8 to 65.4°C after 5 min irradiation, indicating an excellent photothermal effect. To quantitatively assess the photothermal conversion capability of PPANPs, the photothermal conversion efficiency (η) was further determined using a standard heating–cooling method. Under 680 nm laser irradiation (1.0 W cm^−^^2^), a PPANP dispersion (100 µg mL^−^^1^) reached a steady‐state temperature, followed by natural cooling after the laser was switched off (Figure S41a). Linear fitting of the cooling data yielded a thermal time constant (τs) of 157.46 s (R^2^ = 0.994; Figure S41b). Based on the absorbance at 680 nm and the solvent heat contribution, η was calculated to be 49.13%, quantitatively confirming the efficient photothermal conversion of PPANPs. The photothermal antibacterial activity was then estimated by recording the number of clones in plate culture and optical density at 600 nm (OD_600_) for pristine *S. aureus* and *S. aureus* treated with PPANPs@sAb. The *S. aureus* (1 × 10^9^ CFU/mL) was incubated with different concentration of PPANPs@sAb (0, 25, 50, 100 and 200 µg mL^−1^) for 10 min, and the mixture was expose to a 680 nm laser (1 W/cm^2^ for 5 min). Figure 7k shows representative photographs of *S. aureus* colonies after treatment with PPANPs@sAb. Under 680 nm laser irradiation, the number of *S. aureus* colonies decreased markedly with increasing PPANPs@sAb concentration. Without irradiation, the change in *S. aureus* colonies was negligible. The viability of *S. aureus* remained above 89.1% after treatment with PPANPs@sAb alone. In contrast, following incubation with PPANPs@sAb and subsequent 680 nm laser irradiation, the viability of *S. aureus* was significantly reduced (Figure 7l). Figure 7m showed that after exposure to 680 nm laser, the OD_600_ of the PPANPs@sAb and *S. aureus* mixture decreased from 1.369 to 0.354 as the concentration of PPANPs increased (the precipitation of inactivated bacteria, Figure S42). In the absence of 680 nm laser irradiation, OD_600_ of the mixture did not decrease (the absorption of PPANPs led to a slight increase). These results demonstrated the excellent photothermal antibacterial activity of PPANPs against *S. aureus* under low‐power‐density laser irradiation. Furthermore, we observed changes in bacterial morphology and membrane integrity through SEM imaging to confirm the excellent antibacterial effect of PPANPs. As shown in Figure 7n, the *S. aureus* cells treated with PPANPs@sAb (without irradiation) and those exposed to 680 nm laser alone exhibited a smooth and integrated surface, indicating that 680 nm laser radiation and PPANPs@sAb alone had no destructive effect on the bacterial cells. After PPANPs@sAb was treated and exposed to 680 nm laser, the cell walls of *S. aureus* showed obvious fragmentation due to the photothermal effect of PPANPs. These findings confirm that the high temperature generated by PPANPs under low‐power‐density laser radiation could damage the cell wall of *S. aureus* and induce bacterial death, proving the excellent photothermal antibacterial performance. Although PPANPs@sAb was evaluated only against *S. aureus* in this study, photothermal killing may have broader antibacterial potential. Photothermal‐based nanoplatforms have shown activity against multidrug‐resistant ESKAPE pathogens, including Pseudomonas aeruginosa [47], while an AIE‐based photothermal system effectively killed Gram‐positive Streptococcus gordonii and Gram‐negative Porphyromonas gingivalis through membrane damage [48]. However, because PPANPs@sAb relies on an anti‐*S. aureus* antibody for targeting, extension to other joint pathogens would require corresponding recognition ligands or a multiplex targeting strategy. Finally, the in vivo biocompatibility of PPANPs was also evaluated. Healthy mice were intravenously injected with either PBS or PPANPs (2 mg mL^−^^1^, 100 µL), and blood and major organs were collected at day 3 and day 7 post‐injection. Plasma biochemical parameters showed no significant abnormalities compared to the PBS group (Figure S43). Furthermore, H&E staining of major organs revealed no observable tissue damage, necrosis, or inflammatory infiltration, confirming good biocompatibility of PPANPs in vivo (Figure S44). Collectively, these results demonstrate the potential of PPANPs as a multifunctional NIR nanoplatform for in vivo infection imaging, together with strong photothermal antibacterial activity demonstrated in vitro.

## 3 Conclusion

This study successfully reported a universal self‐assembly strategy for constructing ultrabright NIR‐AIEgens fluorescent probes via the co‐assembly of hydrophobic NIR AIEgens, hydrophobic inorganic nanomaterials, and amphiphilic polymers, and revealed the formation mechanism. The core–shell–shell architecture of PPANPs enhanced their fluorescence and photothermal performance through the combined effects of spatial confinement, ligand‐mediated molecular organization, and cooperative luminescence within the PbS–AIE hybrid nanoassembly, thereby enabling their use as integrated nanoprobes for in vitro diagnosis and in vivo imaging. These PPANPs have the potential not only for rapid and ultrasensitive detection of *S. aureus* in complex joint fluid samples but also for early imaging of joint infection, while exhibiting strong photothermal antibacterial activity in vitro under low‐power‐density laser irradiation. Moreover, the consistency of the clinical sample detection results with the plate culture method and the validation of the mouse model of joint infection further demonstrated the potential of PPANPs for clinical application. PPANPs were evaluated only in an *S. aureus* infection model in this study, and additional infection models involving other bacterial species and anatomical sites should be investigated in future studies. Collectively, our research reveals a universal approach for synthesizing ultrabright NIR‐AIEgens biomaterials and provides new opportunities for rapid bacterial diagnosis, in vivo infection imaging, and photothermal antibacterial applications.

## 4 Experimental Section

### 4.1 Materials

Lead oxide (PbO) powder, bis(trimethylsilyl) sulfide (TMS)_2_S, 1‐octadecene (ODE), ammonium hydroxide, n‐hexane, cyclohexane, oleic acid (OA), chloroform, tetraethyl orthosilicate (TEOS), phosphate‐buffered saline (PBS) solution, borate‐buffered saline (BBS) solution, and thioglycolic acid were purchased from Aladdin Biochemical Technology Co., Ltd. (Shanghai, China) and Macklin Biochemical Co., Ltd. (Shanghai, China). Polyvinylpyrrolidone (PVP), nonylphenol ethoxylate (CO‐520), poly(maleic anhydride‐alt‐1‐octadecene) (PMAO), [3‐(trimethoxysilyl)propyl] methacrylate, sodium dodecyl sulfate (SDS), cadmium nitrate tetrahydrate (Cd(NO_3_)_2_·4H_2_O), tellurium (Te) powder, and sodium borohydride (NaBH_4_) were obtained from Sigma–Aldrich (St. Louis, MO, USA). Methanol, acetone, and anhydrous ethanol were supplied by Chongqing Chuandong Chemical Co., Ltd. (Chongqing, China). Nitrogen gas was obtained from Chengdu Hongjin Chemical Co., Ltd. (Chengdu, China). The NC membrane was purchased from PALL Manufacturing UK Limited (Portsmouth, UK). Glass fiber membranes and absorbent pads were obtained from Shanghai Jieyi Biotechnology Co., Ltd. (Shanghai, China). Sodium chloride (NaCl) and sucrose were purchased from Macklin Biochemical Co., Ltd. (Shanghai, China). Tyrosine and casein were supplied by Aladdin Biochemical Technology Co., Ltd. (Shanghai, China). Bovine serum albumin (BSA), CRP‐labeled antibodies, CRP‐coating antibodies, IgY antigen, goat anti‐mouse IgY, and goat anti‐mouse secondary antibody were provided by Guangdong Fapon Biotech Co., Ltd. (Guangdong, China). Tween‐80 was purchased from Thermo Fisher Scientific (Waltham, MA, USA), and tetrakis(ethylene glycol) monolaurate (Thesit) was obtained from Beyotime Biotechnology Co., Ltd. (Shanghai, China). Condenser reflux tubes, 50 mL three‐necked flasks, and soft rubber stoppers for flasks were purchased from Shanghai Anserui Co., Ltd. (Shanghai, China).

### 4.2 Characterization

The UV–vis absorption spectra were measured using a UV–vis spectrophotometer (UH5300, Hitachi, Japan). NIR fluorescence emission spectra were recorded using a modified fluorescence spectrophotometer (F‐4700, Hitachi, Japan). Dynamic light scattering (DLS) and zeta potential measurements were carried out on a nanoparticle size and zeta potential analyzer (Zetasizer Nano ZS90, Malvern Instruments, UK). X‐ray diffraction (XRD) patterns were acquired using an X‐ray diffractometer (Bruker, Germany), and X‐ray photoelectron spectroscopy (XPS) analysis was performed using an XPS system (Shimadzu, Japan). Transmission electron microscopy (TEM) images were obtained using a field‐emission transmission electron microscope (FEI, USA). Photothermal heating was achieved using a 680 nm laser (Huashang Laser Technology Co., Ltd., Shenzhen, China), and temperature monitoring was performed using an infrared thermal imager (FLIR, USA). A 365 nm LED UV lamp (Huashang Laser Technology Co., Ltd., Shenzhen, China) was used to excite visible‐region AIEgens. NIR fluorescence images of lateral flow assay (LFA) test strips were acquired using a self‐developed portable NIR‐LFA imaging platform equipped with an industrial camera and a 720 nm long‐pass filter (MydeVision Technology Co., Ltd., Shenzhen, China). Ultrafast centrifugation was conducted using a high‐speed refrigerated centrifuge (Thermo Fisher Scientific, USA). In vivo fluorescence imaging was performed using an IVIS Lumina Series III small animal imaging system (PerkinElmer, USA). Molecular models were constructed using PACKMOL and Materials Studio software, and all molecular dynamics (MD) simulations were performed using the GROMACS package (version 2019). Visualization and surface analysis of MD models were conducted using Visual Molecular Dynamics (VMD, version 4.0).

### 4.3 Synthesis of OA‐Capped PbS QDs

PbS QDs were synthesized via a modified hot‐injection method. The sulfur precursor was prepared by mixing 0.2 mL of bis(trimethylsilyl) sulfide ((TMS)_2_S) with 5 mL of 1‐octadecene (ODE), followed by degassing under vacuum for 2 h and storage under nitrogen. For the lead precursor, 0.446 g of PbO was added to 20 mL ODE and 1.5 mL oleic acid (OA) in a 100 mL three‐necked flask. After degassing at room temperature for 5 min, the mixture was heated to 120 °C under vacuum for 1 h until fully clarified. The solution was purged with nitrogen and adjusted to the designated injection temperature (80–180 °C). Once stabilized, 5.2 mL of sulfur precursor was rapidly injected under a nitrogen atmosphere, and the reaction proceeded for 2 min. The heating source was then removed, and the solution cooled in an ice bath. The crude product was precipitated with 10 mL acetone, centrifuged at 13,500 rpm and 4 °C for 15 min, redispersed in chloroform, and washed by repeated acetone precipitation and centrifugation (3×). Purified PbS QDs were finally redispersed in 4 mL chloroform and stored at 4 °C.

### 4.4 Preparation of PPANPs

PPANPs were prepared by nanoprecipitation using a gradient of AIE dye concentrations ranging from 0.25 to 2.0 mg. Briefly, 5 mg of PMAO was dissolved in 50 µL of chloroform, and 9 mg of OA‐capped PbS QDs were dispersed in 25 µL of chloroform. AIE dye was dissolved in 50 µL of chloroform and mixed with PbS QDs under ultrasonication. The resulting AIE–PbS mixture was then combined with the PMAO solution under ultrasonic conditions. Each formulation was slowly added dropwise along the wall of a beaker containing 300 mL of SDS solution (0.5 mg mL^−^^1^) to induce interfacial assembly. The mixture was emulsified using a cell disruptor under an ice bath (10% amplitude, 2 min total, 9.9 s on, 5 s off). After emulsification, 2 mL of ultrapure water was added. The organic solvent was removed by rotary evaporation at 37 °C and 800 rpm for 30 min. The resulting emulsion was centrifuged at 12,000 rpm and 4 °C for 15 min. The pellet was redispersed in alkaline water (pH 10) under ultrasonication and allowed to stand for 12 h. After a second centrifugation (12,000 rpm, 15 min), the precipitate was washed and redispersed in ultrapure water and stored at 4 °C until further use.

### 4.5 Optical Characterization of Nanoparticles

For fluorescence spectroscopy, 20 µL of sample was diluted with solvent to a final volume of 2 mL, ultrasonicated to mix, and transferred to a standard quartz fluorescence cuvette. The fluorescence spectrum was recorded after placing the cuvette into the sample holder. The excitation and emission slit widths were both set to 5.0 nm, with a PMT voltage of 700 V. The excitation spectrum was scanned with the emission wavelength fixed at 830 nm to determine the optimal excitation wavelength, which was then used to acquire the emission spectrum. For UV–vis–NIR absorption measurements, two quartz cuvettes were rinsed with ultrapure water and dried. Both were filled with 2 mL of solvent for baseline calibration in the spectrometer (wavelength range: 200–900 nm, scan speed: 1200 nm/min). After calibration, 20 µL of sample was diluted to 2 mL, ultrasonicated, transferred to the sample cuvette, and measured against the reference.

### 4.6 Physicochemical Characterization of PPANPs

The hydrodynamic diameter and zeta potential of AIE_830_@PbS@PMAO nanoparticles (PPANPs) were measured using a nanoparticle size and zeta potential analyser. Briefly, 20 µL of nanoparticle suspension was centrifuged at 13,500 rpm and 4 °C for 15 min, and the pellet was redispersed in 1 mL ultrapure water and stored at 4 °C. Before measurement, the cuvette was rinsed with ultrapure water and dried with lint‐free tissue to remove residual droplets. The nanoparticle suspension was carefully loaded into the cuvette to avoid air bubbles and filled to the indicated line. Measurements were conducted according to standard operating procedures. Zeta potential was measured using a dedicated electrophoretic cell following the same preparation protocol. For TEM imaging, PPANPs were collected by centrifugation (13 500 rpm, 4 °C, 15 min), redispersed in n‐hexane, and ultrasonicated for 5 min. A 1 µL aliquot was dropped onto a carbon‐coated copper grid and dried at room temperature for 30 min to allow solvent evaporation. The morphology of PPANPs was observed under transmission electron microscopy, and representative images were acquired at various magnifications.

### 4.7 Construction of a Laboratory‐Built Portable Miniaturized NIR‐LFA Instrument

A custom‐built portable NIR fluorescence detection system was developed to quantify the test line signal intensity on NIR‐LFA strips. The integrated device consisted of two 680 nm laser diodes with matched output power, a CMOS industrial camera, a built‐in power supply, and optical signal‐processing components, all housed within a closed casing. A card slot was positioned at the bottom of the housing to accommodate the NIR‐LFA test strip. The two laser sources and the CMOS camera were mounted above the slot, with a 720 nm long‐pass filter placed between the test strip and the camera to eliminate visible‐light interference. The optical processor was connected to the CMOS camera to acquire and process the near‐infrared fluorescence signals. The system enabled real‐time output of signal intensities via wired or wireless communication using a dedicated software interface.

### 4.8 Patient Inclusion and Exclusion Criteria

Inclusion criteria: Patients who underwent revision total joint arthroplasty (TJA) at the First Affiliated Hospital of Chongqing Medical University between 2021 and 2023 because of suspected prosthetic joint infection (PJI) or aseptic failure were eligible for inclusion. Only patients with complete clinical information and sufficient synovial fluid samples for microbiological analysis were enrolled.

Exclusion criteria: Patients who had received systemic antibiotic therapy before synovial fluid aspiration or had concomitant infections at other anatomical sites were excluded from the study.

A total of 52 patients meeting the above criteria were initially enrolled. Four *S. aureus*‐negative infection samples were subsequently excluded from the NIR‐LFA analysis because excessive synovial tissue fragments were obtained during sampling, resulting in insufficient recoverable synovial fluid for reliable chromatographic migration. Therefore, 48 samples were included in the final clinical analysis, comprising 12 *S. aureus*‐positive infections, 14 non‐*S. aureus* infections, and 22 aseptic revisions.

### 4.9 Clinical Collection of Synovial Fluid Samples

Synovial fluid was collected from patients experiencing total joint arthroplasty (TJA) failure prior to the initiation of antibiotic therapy on the first day of admission. Joint aspiration was performed under aseptic conditions. The puncture site was marked using an oil‐based pen, either at the suprapatellar region through the lateral quadriceps approach or the parapatellar ligament approach. After skin disinfection, the operator wore sterile gloves and placed sterile drapes. Local anesthesia was administered along the puncture path from the skin to the joint capsule using lidocaine or 2% procaine. A sterile syringe was inserted along the anesthetized path until the joint cavity was reached. Fluid was gently aspirated while stabilizing the needle and syringe. In cases of obstruction, a small amount of air was injected to relieve the blockage before resuming aspiration. The collected fluid was immediately transferred to sterile cryogenic tubes and placed in a 4 °C transport box. Patient identity and hospitalization number were recorded, and the samples were stored at −80 °C. Sample collection was approved by the Ethics Committee of Chongqing Medical University (Chongqing, Permit No. KX2025‐KYC0648‐01).

### 4.10 Preparation of PPANPs@Ab Conjugation

The PPANPs@Ab conjugate was synthesized using the EDC–NHS coupling method. Briefly, 1 mg of PPANPs was centrifuged at 13 000 rpm for 10 min, and the pellet was air‐dried and resuspended in 250 µL of BBS buffer (pH 6.0), followed by ultrasonication in an ice bath for 2 min at 60 W. Then, 25 µL of sulfo‐NHS solution (10 mg mL^−^^1^ in BBS, pH 6.0) and 25 µL of EDC solution (10 mg mL^−^^1^ in BBS, pH 6.0) were added dropwise. The mixture was stirred at room temperature for 30 min. After activation, the PPANPs were centrifuged and resuspended in 300 µL of BBS buffer (pH 7.4), followed by 3 min of ultrasonication in an ice bath. Next, 70 µg of target antibody was added, and the mixture was stirred at room temperature for 3 h to form the PPANPs@Ab conjugate. Casein (dissolved in BBS, pH 8.4) was added to a final concentration of 3 mg mL^−^^1^ for blocking, and the mixture was stirred for 1 h at room temperature. Subsequently, BSA (dissolved in BBS, pH 7.4) was added to a final concentration of 10 mg mL^−^^1^, and blocking continued for an additional 1 h. The final conjugate was centrifuged and resuspended in 500 µL of 2 mg mL^−^^1^ BSA solution (in BBS, pH 7.4), ultrasonicated for 5 min in an ice bath, and stored at 4 °C until use.

### 4.11 Fabrication of PPANPs‐Based LFIA Test Strip

The test strip consisted of five components: a sample pad, a conjugation pad, an NC membrane, an absorbent pad, and a PVC backing plate. On the NC membrane, Hla antigen (0.5 mg mL^−^^1^) or CRP detection antibody (1 mg mL^−^^1^), both prediluted in PBS (pH 6.8, containing 4% sucrose and 4% NaCl), were applied to the T lines for the detection of *S. aureus* and CRP, respectively. For CRP and *S. aureus* detection, goat anti‐mouse IgG (1 mg mL^−^^1^) or goat anti‐chicken IgY (1 mg mL^−^^1^) prediluted in PBS was dispensed on the C lines, respectively. After assembly, the test strips were cut to a width of 3.90 mm and affixed onto the PVC backing plate for subsequent experiments.

### 4.12 Bacterial Culture of Synovial Fluid Samples

Under sterile conditions, 10 µL of synovial fluid was dropped onto the center of a sterile tryptic soy agar (TSA) plate pre‐equilibrated to 4 °C. The sample was evenly spread using a sterile L‐shaped spreader. The plates were air‐dried at room temperature in a sterile environment and then inverted and incubated at 37 °C for 24 h. Colony growth was recorded and matched to the corresponding clinical sample ID. Plates without visible colonies were further incubated for an additional 72 h and re‐evaluated. For samples that remained culture‐negative, the stored synovial fluid was thawed in a 37 °C water bath until fully liquefied and then subjected to ultrasonication (2 mm probe, 30% amplitude, ice bath, 1 min). The sonicated samples were replated onto TSA using the same procedure and incubated at 37 °C for another 24 h. Plates were again assessed for colony formation. Culture‐negative plates were incubated for an additional 72 h before final recording.

### 4.13 Evaluation of Photothermal Effect of PPANPs

The photothermal conversion capacity of PPANPs at different concentrations was evaluated under 680 nm laser irradiation. Aqueous suspensions of PPANPs at concentrations of 0, 25, 50, 100, and 200 µg mL^−^^1^ were irradiated with a 680 nm laser at a power density of 1.0 W cm^−^^2^ for 5 min. During irradiation, the temperature of each sample was recorded using an infrared thermal imager, and infrared thermal images of the sample tubes were captured at the end of the irradiation period.

### 4.14 Photothermal Conversion Efficiency of PPANPs

An aqueous PPANP dispersion (100 µg mL^−^^1^, 200 µL) was irradiated with a 680 nm laser at a power density of 1.0 W cm^−^^2^ for 1200 s until the temperature approached a steady state. The laser was then switched off, and the sample was allowed to cool naturally for 1200 s. The temperature was recorded at 30 s intervals using an infrared thermal imager. The dimensionless temperature parameter θ was calculated according to θ = (T − T_surr_)/(T_max_ − T_surr_), where T, T_surr_, and T_max_ represent the sample temperature at a given time, the ambient temperature, and the maximum steady‐state temperature, respectively. The thermal time constant (τ_s_) was obtained from linear fitting of cooling time against −ln(θ). The photothermal conversion efficiency was calculated according to η = [hS(T_max_ − T_surr_) − Qdis]/[P(1 − $10^{-A_{680}}$)] × 100%, where hS = mC_p_/τ_s_, P is the incident laser power, A_680_ is the absorbance of PPANPs at 680 nm, and Qdis represents the background heat contribution from the solvent and sample container under identical irradiation conditions.

### 4.15 In Vitro Photothermal Antibacterial Assay

To evaluate the photothermal antibacterial efficacy of PPANPs, *S. aureus* suspensions at OD_600_  =  1.0 (∼1 × 10^8^ CFU mL^−^^1^) were incubated with PPANPs at a final concentration of 200 µg mL^−^^1^. The mixtures were gently shaken at 37 °C for 30 min to allow sufficient interaction between the nanoparticles and bacterial cells. A 680 nm laser (1.0 W cm^−^^2^) was then applied vertically for 5 min at a fixed distance to ensure uniform illumination. Four groups were included in the experiment: treatment with PPANPs under laser irradiation, treatment with PPANPs without laser exposure, laser exposure alone, and untreated control. After treatment, antibacterial efficacy was evaluated using TEM imaging to observe bacterial membrane disruption, OD_600_ measurements to monitor bacterial proliferation, and colony formation analysis by plating 10 µL of each sample on TSA followed by incubation at 37 °C for 24 h. Colony numbers were manually counted to quantify bacterial survival. All experiments were performed in triplicate to ensure reproducibility.

### 4.16 *S. Aureus*‐Induced Mouse Model of Joint Infection and In Vivo Nir Fluorescence Imaging

Female C57BL/6 mice (6–8 weeks old, 25–30 g) were purchased from the Experimental Animal Center of Chongqing Medical University and maintained under specific pathogen‐free (SPF) conditions with a 12‐h light–dark cycle, temperature of 20–22 °C, and humidity of 30%–70%. All animal experiments were performed in accordance with the International Guide for the Care and Use of Laboratory Animals and approved by the Research Ethics Committee of the Third Military Medical University (Chongqing, Permit No. 2011–04). A periprosthetic joint infection model was established by intra‐articular injection of *S. aureus* (ATCC 29213) into the mouse hip joint (10 µL, 1 × 10^8^ CFU mL^−^^1^). Prior to surgery, mice were anesthetized with isoflurane (4%–5% for induction, 1%–2% for maintenance), and the surgical site was disinfected with sterile saline. At 24 h post‐infection, 100 µL of PPANPs‐Ab (2 mg mL^−^^1^) was administered via tail vein injection, corresponding to a dosage of approximately 6.67 mg kg^−^^1^. At predetermined time points after injection, in vivo NIR fluorescence imaging was performed using a laboratory‐built portable system equipped with a 680 nm excitation laser and a 720 nm long‐pass filter. The fluorescence intensity at the infection site and major organs was recorded, and quantitative analysis was performed using ImageJ software.

### 4.17 In Vitro Photothermal Antibacterial Assay

To evaluate the photothermal antibacterial efficacy of PPANPs, *S. aureus* suspensions at OD_600_  =  1.0 (∼1 × 10^8^ CFU mL^−^^1^) were incubated with PPANPs at a final concentration of 200 µg mL^−^^1^. The mixtures were gently shaken at 37 °C for 30 min to allow sufficient interaction between the nanoparticles and bacterial cells. A 680 nm laser (1.0 W cm^−^^2^) was then applied vertically for 10 min at a fixed distance to ensure uniform illumination. Four groups were included in the experiment: treatment with PPANPs under laser irradiation, treatment with PPANPs without laser exposure, laser exposure alone, and untreated control. After treatment, antibacterial efficacy was evaluated using TEM imaging to observe bacterial membrane disruption, OD_600_ measurements to monitor bacterial proliferation, and colony formation analysis by plating 10 µL of each sample on TSA followed by incubation at 37 °C for 24 h. Colony numbers were manually counted to quantify bacterial survival. All experiments were performed in triplicate to ensure reproducibility.

### 4.18 Data Analysis

All statistical analyses were performed using GraphPad Prism 9 and Origin 64 software. Quantitative data were presented as mean ± standard deviation (mean ± SD). Differences between two groups were assessed using two‐sided unpaired Student's t‐tests. For comparisons among multiple groups, one‐way analysis of variance (one‐way ANOVA) followed by Tukey's multiple comparisons test was applied. A P value less than 0.05 was considered statistically significant.

## Author Contributions

**Yujian Li**: methodology, investigation, validation, Writing – original draft, Writing – review and editing. **Jia Shu**: software, investigation. **Sijia Lv**: writing – original draft, validation. **Leilei Qin**: formal analysis, supervision. **Xuefeng Luo**: software. **PengCheng Xiao**: data curation. **Huan Cai**: data curation. **Xin Gao**: investigation. **Yan Zhao**: data curation. **Haotian Zhou**: formal analysis. **Yaji Yang**: visualization. **Zhenglin Zhu**: project administration. **Shijia Ding**: funding acquisition. **Juan Li**: funding acquisition, supervision, methodology. **Rui Chen**: writing – review and editing, conceptualization, project administration. **Wei Huang**: project administration, conceptualization, resources, writing – review and editing.

## Funding

This work was supported by the National Natural Science Foundation of China (82302646, 82402836, 82272432), the Regional Innovation and Development Joint Fund of the National Natural Science Foundation of China (U22A20284), and the Discipline Excellence Program of the First Affiliated Hospital of Chongqing Medical University (cyyy‐xkdfjh‐lcyj‐202304). The authors also acknowledge the support from the Innovation and Development Joint Fund of Chongqing Natural Science Foundation (CSTB2024NSCQ‐LZX0058), the Chongqing Medical University International Institute of In Vitro Diagnostics Residency Program (IVD20231001, IVD2025rzxm‐07), The “Digital‐Intelligent Medical Laboratory Testing” Open Competition Project (WDIM2025JBGS‐SZJY001, WDIM2025JBGS‐SZJY008), and Chongqing Higher Education Teaching Reform Research Project (No. 256040).

## Conflicts of Interest

The authors declare no conflicts of interest.

## Supporting information




**Supporting File 1**: advs78156‐sup‐0001‐SuppMat.docx.

**Supporting File 2**: advs78156‐sup‐0002‐VideoS1.mp4.

**Supporting File 3**: advs78156‐sup‐0001‐VideoS2.mp4.

**Supporting File 4**: advs78156‐sup‐0001‐VideoS3.mp4.

## Data Availability

The data that supports the findings of this study are available in the supplementary material of this article.
