# Supplementary material for: Spatially Confined/Ligand‐Directed Ultrabright NIR‐AIE Nanoprobe for In Vitro Rapid Detection and In Situ Imaging of Bacterial Septic Arthritis
Source: Adv Sci (Weinh). 2026 Oct 6:e78156. Online ahead of print. doi: 10.1002/advs.78156 (PMC13643110; doi:10.1002/advs.78156)
Supplement: Supplementary file 1 [file ADVS-9999-e78156-s003.docx]

Supporting Information

Spatially Confined/Ligand-Directed Ultrabright NIR-AIE Nanoprobe for In Vitro Rapid Detection and In Situ Imaging of Bacterial Septic Arthritis

Yujian Li, Jia Shu, Sijia Lv, Leilei Qin, Xuefeng Luo, PengCheng Xiao, Huan Cai, Xin Gao, Yan Zhao, Haotian Zhou, Yaji Yang, Zhenglin Zhu, Shijia Ding, Juan Li*, Rui Chen*, and Wei Huang*

**Figure S1.** Molecular formula of AIE.

**Figure S2.** UV absorption spectrum and fluorescence spectrum (λex=380nm) of PbS QDs in chloroform.

**Figure S3.** TEM image of PbS QDs with an inset showing a high-magnification image.

**Figure S4. Characterization of PMAO@PbS nanoparticles.** (a-c) TEM images of PbS QDs coated with PMAO at different masses (from left to right: 6mg, 9mg, 12mg). Insets show the physical photos of the solutions. d) HAADF STEM and EDS spectral analysis of PMAO@PbS.

**Figure S5.** Hydrodynamic diameter (a) and zeta potential (b) of PMAO-encapsulated PbS QDs at different input masses (6, 9, and 12 mg).

**Figure S6.** Fluorescence (a) and UV–vis absorption (b) spectra of PMAO-encapsulated PbS QDs at different input masses (6, 9, and 12 mg).

**Figure S7.** Dispersion characterization of PMAO-encapsulated AIE nanoparticles at different input masses (1.5 and 9 mg). a, Hydrodynamic diameter. b, Zeta potential.

**Figure S8.** Photographs and TEM images of PMAO@PbS (12 mg, 242 nm) after storing for 12 hours, with red arrows indicating PMAO failed to encapsulate PbS QDs on the surface.

**Figure S9.** DLS-measured hydrodynamic size distribution of PPANPs with different AIEgen loadings.

**Figure S10.** Zeta-potential analysis of PPANPs with different AIEgen loadings.

**Figure S11.** TEM images, HAADF-STEM images, and line-scan elemental profiles (C, N, Pb, and S) of PMAO@PbS (a) and PPANPs (b).

**Figure S12.** Nanoparticle concentration analysis of different formulations measured by flow cytometry. a, Particle concentration of PMAO@PbS. b, Particle concentration of PMAO@AIE (1.5 mg). c, Particle concentration of PMAO@AIE (9 mg). d, Particle concentration of PPANPs.

**Figure S13.** Fluorescence and UV–vis absorption spectra of PPANPs and PMAO-encapsulated nanoparticles containing either pure PbS QDs (PMAO@PbS) or AIEgens (PMAO@AIE).

**Figure S14.** Scanning electron microscopy (SEM) image of PS@AIE nanoparticles.

**Figure S15.** Quantum yield (QY) quantification of PPANPs in aqueous solution. a, UV–vis–NIR absorption spectra of ICG in water at increasing concentrations (0.25, 0.5, 1.0, 2.0, and 4.0 μg mL⁻¹). b, Fluorescence spectra of ICG in water under 630 nm laser excitation (1 W cm⁻²) with the same concentration gradient. c, Integrated fluorescence intensity plotted as a function of optical density (OD) at 630 nm for ICG, based on data from (a) and (b). d, UV–vis–NIR absorption spectra of PPANPs in water at increasing concentrations (2.5, 5.0, 10.0, 20.0, and 40.0 μg mL⁻¹). e, Fluorescence spectra of PPANPs under 630 nm laser excitation (1 W cm⁻²) across the same concentration range. f, Integrated fluorescence intensity plotted as a function of OD at 630 nm for PPANPs, based on data from (d) and (e). The QY of PPANPs was calculated to be 16.4%.

**Figure S16.** XRD spectra of PPANPs, PPNPs, and PANPs.

**Figure S17.** Photobleaching resistance test of PPANPs and ICG under continuous irradiation with a 680 nm laser (1 W cm⁻²) for 1 hour.

**Figure S18.** Thermal stability test of PPANPs and ICG (in a constant temperature drying oven at 45°C).

**Figure S19.** Fluorescence stability of PPANPs in different solvents.

**Figure S20.** Fluorescence stability of PPANPs under different pH conditions.

**Figure S21.** Hydrodynamic diameter changes of PPNPs stored at room temperature for one week.

**Figure S22.** Zeta potential changes of PPNPs stored at room temperature for one week.

**Figure S23.** Comparison of fluorescence intensity AUC of PPANPs after two months of storage.

**Figure S24. Fluorescence stability of PPANPs under H₂O₂-mimicked oxidative conditions.** a, Representative photographs of PPANP suspensions before and after incubation with different concentrations of H₂O₂ (0, 100 μM, and 1 mM) at 37 °C for 24 h. b, Fluorescence retention of PPANPs after 24 h incubation under the corresponding conditions. Fluorescence retention was calculated as $I_{24h}/I_{0h}\times100\%$. Data are presented as mean ± SD (n = 3).

**Figure S25.** Interaction forces between AIE molecules in different models. a,Lennard-Jones (SR) interaction forces between AIE molecules. b, Coulomb (SR) interaction forces between AIE molecules.

**Figure S26.** a, 3D schematic diagram of the AIE molecule highlighting the side-chain dihedral angle formed by four carbon atoms (indicated by the red circle and arrow). b, Dihedral angle distribution of the AIE molecule.

**Figure S27.** Validation of the portable integrated NIR fluorescence detection instrument. a, Integrated fluorescence intensity (area under the curve) of different nanoparticle solutions measured at equal particle concentrations using the custom-built NIR device; insets show photographs of the corresponding solutions. b, Representative NIR fluorescence images of different nanoparticle probes on lateral flow strips, captured using the CMOS industrial camera.

**Figure S28.** Photographs of visible-light quantum dots under daylight and UV light (excitation wavelength at 365 nm). From left to right: green QDs (emission at 530 nm), yellow QDs (580 nm), and red QDs (630 nm).

**Figure S29.** Optimization of immunoreaction time for NIR-LFA strips. a, Test strip images captured by the camera of the NIR-LFA instrument. b, Test line/control line signal ratios (It/Ic) for Hla protein samples and negative samples at different immunoreaction times. c, Ratio of It/Ic for Hla samples (Sh) to negative samples (Nh) under different immunoreaction times (Sh/Nh).

**Figure S30.** Optimization of PPANPs@Ab material concentration for NIR-LFA strips. a, Test strip images captured by the NIR-LFA instrument camera. b, Test/control line signal ratios (It/Ic) for Hla protein samples and negative samples at different concentrations of PPANPs@Ab. c, Sh/Nh values under different PPANPs@Ab concentrations, representing the ratio of It/Ic for Hla samples (Sh) to that for negative samples (Nh).

**Figure S31.** Optimization of exposure index for NIR-LFA strips. a, Test strip images captured by the NIR-LFA instrument camera. b, Test/control line signal ratios (It/Ic) for Hla protein samples and negative samples under different exposure index settings. c, Sh/Nh values under different exposure index settings, representing the ratio of It/Ic for Hla samples (Sh) to that for negative samples (Nh).

**Figure S32.** Optimization of HLA protein immobilized concentration on the T line for NIR-LFA strips. a, Test strip images captured by the NIR-LFA instrument camera. b, Test/control line signal ratios (It/Ic) for Hla-positive samples and negative samples at different HLA protein immobilized concentrations. c, Sh/Nh values under different HLA protein immobilized concentrations, representing the ratio of It/Ic for Hla samples (Sh) to that for negative samples (Nh).

**Figure S33.** PPANPs-LFA discrimination of *S. aureus* (n = 12) and non-*S. aureus* (n = 36) synovial fluid samples in the validation cohort. Dashed line indicates the cutoff value.

**Figure S34.** Bacterial culture results of 12 synovial fluid samples positive for *S. aureus* infection.

**Figure S35.** Optimization of immunoreaction time for CRP protein detection using NIR-LFA strips. a, Test strip images captured by the NIR-LFA instrument camera. b, Test/control line signal ratios (It/Ic) for CRP-positive and negative samples at different immunoreaction times.

**Figure S36.** Optimization of PPANPs@Ab material concentration for CRP protein detection using NIR-LFA strips. a, Test strip images captured by the NIR-LFA instrument camera. b, Test/control line signal ratios (It/Ic) for CRP-positive and negative samples with different concentrations of PPANPs@Ab. c, Sc/Nc values representing the ratio of It/Ic for CRP-positive samples (Sc) to that for negative samples (Nc) at different concentrations of PPANPs@Ab.

**Figure S37.** Optimization of exposure index for CRP protein detection using NIR-LFA strips. a, Test strip images captured by the NIR-LFA instrument camera. b, Test/control line signal ratios (It/Ic) for CRP-positive and negative samples at different exposure indices. c, Sc/Nc values representing the ratio of It/Ic for CRP-positive samples (Sc) to that for negative samples (Nc) at different exposure indices.

**Figure S38.** a, Fluorescence images of different concentrations of CRP protein detected on the NC membrane, captured using the portable miniaturized NIR-LFA instrument. b, CRP sensitivity curve constructed using NIR-LFA.

**Figure S39.** Correlation analysis between PPANPs-LFA and clinical CRP detection.

**Figure S40.** a, DLS-measured hydrodynamic diameter of PPANPs before and after incubation with sAb. b, Zeta potential of PPANPs before and after incubation with sAb.

**Figure S41. Photothermal conversion efficiency of PPANPs.** a, Photothermal heating and natural cooling curve of PPANPs (100 μg mL⁻¹) under 680 nm laser irradiation at 1.0 W cm⁻². The laser was switched off after 1200 s irradiation. b, Linear fitting of cooling time versus −ln(θ) for determination of the thermal time constant (τs). The calculated photothermal conversion efficiency (η) of PPANPs was 49.13%.

**Figure S42.** Precipitation of *S. aureus* from solution observed after 5min of 680 nm laser irradiation in the presence of PPANPs@sAb.

**Figure S43.** Plasma biochemical indicators were measured in healthy mice at 3 or 7 days after intravenous injection of 100 μL of PPANPs (2 mg mL⁻¹) or PBS. n = 5 mice in each group. ALT, alanine transaminase; AST, aspartate transaminase; ALB, albumin; TBA, total bile acid; BUN, blood urea nitrogen; CRE, creatinine. All error bars indicate mean ± SD.

**Figure S44.** H&E staining of major organs/tissues from healthy mice at 3 or 7 days after intravenous injection of 100 μL of PPANPs (2 mg mL⁻¹). Scale bar = 100 μm. Experiments were independently repeated five times with consistent results; representative images are shown.

**Table S1.** Representative NIR-AIE nanoprobes and their quantum yields.

| **Nanoprobe** | **Assembly strategy** | **Emission region** | **QY** | **Reference** |
| --- | --- | --- | --- | --- |
| PS@AIE₈₃₀NPs (previously reported by our group) | Polymer-supported NIR-AIE nanoparticle assembly | NIR-I | 14.76% | Ref. 26 |
| α-DTPEBBTD-C4 NPs | Amphiphilic block copolymer-assisted assembly | NIR-I  (800–815 nm) | up to 4.8% | Ref. 27 |
| PEG-TTB AIEgen NPs | Molecular self-assembly | NIR-II | ~10.3% | Ref. 28 |
| TTB NPs | Molecular and morphological modulation | NIR-II | ~11.5% | Ref. 29 |
| **PPANPs** | **PbS-confined core–shell–shell co-assembly strategy** | **NIR-I** | **16.40%** | **This work** |

**Table S2.** Demographic and clinical characteristics of patients enrolled in the study.

| **Demographic characteristics** | **PJI(n=26)** | | **Aseptic revision**  **(n=22)** |
| --- | --- | --- | --- |
|  | **S. aureus infection**  **(n=12)** | **Non-S. aureus. Infection**  **(n=14)** |  |
| Age(SD) | 68.36(14.43) | 73.41(13.96) | 72.53(15.04) |
| BMI (SD) | 22.37(4.25) | 22.64(3.39) | 23.86(4.82) |
| SEX (%) |  |  |  |
| male | 6(50.00) | 5(35.71) | 12(54.55) |
| female | 6(50.00) | 9(64.29) | 10(45.45) |
| Joint Type(%) |  |  |  |
| Knee joint | 4(33.33) | 8(57.14) | 13(59.09) |
| Hip joint | 8(66.67) | 6(42.86) | 9(40.91) |

**Table S3.** The Musculoskeletal Society 2018 definition of prosthetic joint infection.

| **diagnostic  criteria** | **MSIS definition of PJI** |  | **Decision** |
| --- | --- | --- | --- |
| major criteria | Two positive cultures of the same organism | | Infected* |
|  | Sinus tract with evidence of communication to  the joint or visualization of the prosthesis | |  |
| Minor criteria | preoperative Diagnosis | Score | Minor criteria  Serum  Synovial |
| Serum | Elevated CRP or D-Dimer | 2 |  |
|  | Elevated ESR | 1 |  |
| Synovial | Elevated synovial WBC count or LE | 3 |  |
|  | Positive alpha-defension | 3 |  |
|  | Elevated synovial PMN(%) | 2 |  |
|  | Elevated synovial CRP | 1 |  |

*One of the major criteria must be met for diagnosis of PJI. CRP, C-reactive protein; ESR, erythrocyte sedimentation rate; LE, leukocyte esterase; PMN, polymorphonuclear; WBC, white blood cell.

**Table S4.** **Diagnostic contingency table of the PPANPs-LFA for clinical detection of *S. aureus*.**

| **PPANPs-LFA result** | ***S. aureus*-positive** | ***S. aureus*-negative** | **Total** |
| --- | --- | --- | --- |
| **Positive** | 12 (TP) | 0 (FP) | 12 |
| **Negative** | 0 (FN) | 36 (TN) | 36 |
| **Total** | 12 | 36 | 48 |

TP, true positive; TN, true negative; FP, false positive; FN, false negative. The sensitivity, specificity, positive predictive value (PPV), negative predictive value (NPV), and overall accuracy were all 100% based on 48 clinical synovial fluid samples, including 12 *S. aureus*-positive and 36 *S. aureus*-negative samples.

**Video S1. Molecular dynamics simulation of Model 1 (PbS limit group).**
Trajectory of 100 AIE molecules over a 100-ns simulation in the PbS confinement model, showing the spatial restriction effect of PbS on AIE molecular motion and packing.

**Video S2. Molecular dynamics simulation of Model 2 (PMAO-encapsulated group without PbS confinement).**
Trajectory of 100 AIE molecules over a 100-ns simulation with PMAO encapsulation but without PbS, illustrating partial spatial restriction from the polymer matrix and intermediate packing order.

**Video S3. Molecular dynamics simulation of Model 3 (control group).**
Trajectory of 100 AIE molecules over a 100-ns simulation in the control model, representing aseline molecular dynamics without spatial confinement or PbS interaction.
